# Efficacy and safety of bevacizumab plus chemotherapy compared to chemotherapy alone in previously untreated advanced or metastatic colorectal cancer: a systematic review and meta-analysis

**DOI:** 10.1186/s12885-016-2734-y

**Published:** 2016-08-24

**Authors:** Tobias Engel Ayer Botrel, Luciana Gontijo de Oliveira Clark, Luciano Paladini, Otávio Augusto C. Clark

**Affiliations:** 1 Evidencias - A Kantar Health Company, Av. José de Souza Campos, 550 - 7°. andar (salas 71 e 72), Nova Campinas, Campinas, São Paulo Brazil 13092-123; 2 CIOP - Centro Integrado de Oncologia e Pesquisa, Rua Santo Antônio 200, sala 301, Poços de Caldas, Minas Gerais Brazil 37701-036

**Keywords:** Chemotherapy, Bevacizumab, Metastatic colorectal cancer, Systematic review, Meta-analysis

## Abstract

**Background:**

Colorectal cancer (CRC) is the fourth most frequently diagnosed cancer and the second leading cause of neoplasm-related death in the United States. Several studies analyzed the efficacy of bevacizumab combined with different chemotherapy regimens consisting on drugs such as 5-FU, capecitabine, irinotecan and oxaliplatin. This systematic review aims to evaluate the effectiveness and safety of chemotherapy plus bevacizumab versus chemotherapy alone in patients with previously untreated advanced or metastatic colorectal cancer (mCRC).

**Methods:**

Several databases were searched, including MEDLINE, EMBASE, LILACS, and CENTRAL. The primary endpoints were overall survival and progression-free survival. Data extracted from the studies were combined by using hazard ratio (HR) or risk ratio (RR) with their corresponding 95 % confidence intervals (95 % CI).

**Results:**

The final analysis included 9 trials comprising 3,914 patients. Patients who received the combined treatment (chemotherapy + bevacizumab) had higher response rates (RR = 0.89; 95 % CI: 0.82 to 0.96; *p* = 0.003) with heterogeneity, higher progression-free survival (HR = 0.69; 95 % CI: 0.63 to 0.75; *p* < 0.00001) and also higher overall survival rates (HR = 0.87; 95 % CI: 0.80 to 0.95; *p* = 0.002) with moderate heterogeneity. Regarding adverse events and severe toxicities (grade ≥ 3), the group receiving the combined therapy had higher rates of hypertension (RR = 3.56 95 % CI: 2.58 to 4.92; *p* < 0.00001), proteinuria (RR = 1.89; 95 % CI: 1.26 to 2.84; *p* = 0.002), gastrointestinal perforation (RR = 3.63; 95 % CI: 1.31 to 10.09; *p* = 0.01), any thromboembolic events (RR = 1.44; 95 % CI: 1.20 to 1.73; *p* = 0.0001), and bleeding (RR = 1.81; 95 % CI: 1.22 to 2.67; *p* = 0.003).

**Conclusion:**

The combination of chemotherapy with bevacizumab increased the response rate, progression-free survival and overall survival of patients with mCRC without prior chemotherapy. The results of progression-free survival (PFS) and overall survival (OS) were comparatively higher in those subgroups of patients receiving bolus 5-FU or capecitabine-based chemotherapy plus bevacizumab, when compared to patients treated with infusional %-FU plus bevacizumab (no difference in PFS and OS). Regarding the type of cytotoxic scheme, regimens containing irinotecan and fluoropyrimidine monotherapy showed superior efficacy results when combined to bevacizumab.

**Electronic supplementary material:**

The online version of this article (doi:10.1186/s12885-016-2734-y) contains supplementary material, which is available to authorized users.

## Background

Colorectal cancer (CRC) is the fourth most frequently diagnosed cancer and the second leading cause of neoplasm-related death in the United States [1, 2]. Thus, CRC constitutes a public health problem that affects men and women in similar proportion and is more prevalent in Western countries [3].

Approximately 25 % of patients already have metastatic disease at the moment of diagnosis and nearly 50 % will develop metastases [4, 5]. Over the past 10 years, various combinations of chemotherapy were investigated for the treatment of metastatic colorectal cancer (mCRC) [6].

Since its introduction by Heidelberger in 1957, 5-fluorouracil (5-FU) has become one of the most extensively used drugs in the treatment of mCRC worldwide and also the backbone of nearly all the recommended and researched chemotherapy associations [7–9]. Capecitabine, another oral fluoropyrimidine, is currently recommended as an alternative for the treatment of these patients since its similar efficacy to 5-FU was demonstrated in randomized studies [10]. Subsequently, two other cytotoxic drugs (irinotecan and oxaliplatin) had their efficacy confirmed in the treatment of mCRC, thus becoming part of treatment protocols since 1999 [7, 11–13].

Lately, the eyes of the medical community around the world have been turned to targeted molecular therapies. In February 2004, the Food and Drug Administration (FDA) approved bevacizumab - a recombinant humanized monoclonal antibody against vascular endothelial growth factor (VEGF) - combined with standard chemotherapy to treat mCRC [14, 15]. Less than a year later, the European Medicines Agency (EMEA) also gave the drug its approval.

Several studies analyzed the efficacy of bevacizumab combined with different chemotherapy regimens consisting on drugs such as 5-FU, capecitabine, irinotecan and oxaliplatin [16]. Four meta-analyses published between 2009 and 2012 compiled the results of randomized trials on standard chemotherapy with bevacizumab in the therapy of mCRC [16–19]. Results of these meta-analyses evidenced the difference in overall survival favoring the groups treated with chemotherapy plus bevacizumab. In 2014, another meta-analysis [20] showed that the addition of bevacizumab to first-line chemotherapy for mCRC did not achieve clinical benefit for overall survival. This latter study brought out questions about the real benefit of chemotherapy plus bevacizumab for these patients. Since then, the availability of new clinical studies produced uncertain or controversial results regarding the effectiveness of this treatment, particularly for the endpoint of overall survival [21–24]. In this context, we felt it was appropriate to re-assess the role of bevacizumab as a component of first-line therapy in patients with advanced colorectal cancer.

This systematic review aims to evaluate the effectiveness and safety of bevacizumab associated with standard chemotherapy in the treatment of patients with mCRC without prior chemotherapy.

## Methods

### Study selection criteria

#### Types of studies

Randomized controlled clinical trials (RCTs) with parallel design that compared the use of chemotherapy regimens associated with bevacizumab against other regimens without bevacizumab.

#### Types of participants

Patients aged ≥ 18 years old with cytological or histological diagnosis of mCRC without prior chemotherapy (only in first-line treatment).

### Search strategy for identification of studies

A wide search of the main computerized databases of interest was conducted, including EMBASE, LILACS, MEDLINE, SCI, CENTRAL, The National Cancer Institute Clinical Trials service, and The Clinical Trials Register. In addition, the abstracts published in the proceedings of the American Society of Clinical Oncology (ASCO), American Association for Cancer Research (AACR), European Society for Medical Oncology (ESMO) and World Congress on Gastrointestinal Cancer were also searched.

For MEDLINE, we used the search strategy methodology for randomized controlled trials [25] recommended by the Cochrane Collaboration [26]. For EMBASE, we used adaptations of this same strategy [25], and for LILACS, we used the search strategy methodology reported by Castro et al. [27]. We performed an additional search on the SCI database looking for papers that were cited on the included studies. We added the specific terms pertinent to this review to the overall search strategy methodology for each database.

The overall search strategy was: #1 “bevacizumab” (Supplementary Concept) OR “bevacizumab” (All Fields); 2# “colorectal neoplasms” (MeSH Terms) OR “colorectal” (All Fields) AND “neoplasms” (All Fields) OR “colorectal neoplasms” (All Fields) OR “colorectal” (All Fields) AND “cancer” (All Fields) OR “colorectal cancer” (All Fields); 3# Clinical Trial (ptyp).

Searches of electronic databases combined the terms #1 AND #2 AND #3 and did not have language or date restrictions.

### Critical evaluation of the selected studies

All the references retrieved by the search strategies had their title and abstract evaluated by two of the researchers. Every reference with the least indication of fulfilling the inclusion criteria was listed as pre-selected. We retrieved the complete article of all pre-selected references. Two different researchers analyzed the articles and included or excluded them according to the previously reported criteria. The excluded trials and the reason for their exclusion are listed in this article. Data was extracted from all the included trials.

Details regarding the main methodology characteristics empirically linked to bias [28] were extracted with the methodological validity of each selected trial assessed by two reviewers (T.E.A.B and O.C). Particular attention was given to some items such as: the generation and concealment of the sequence of randomization, blinding, application of intention-to-treat analysis, sample size pre-definition, loss of follow-up description, adverse events reports, if the trial was multicentric and the source of sponsorship.

### Data extraction

Two independent reviewers extracted the data. The name of the first author and year of publication were used to identify the study. All data were extracted directly from the text or calculated from the available information when necessary. The data of all trials were based on the intention-to-treat principle, so they compared all patients allocated in one treatment with all those allocated in the other arm.

The primary endpoints were progression-free survival (defined as time from randomization to either death or disease progression, whichever occurred first) and overall survival. If data on progression-free survival were not available, data on time to progression or event free survival were assessed.

Other clinical outcomes were evaluated: overall response rate (complete response + partial response) and the more frequently found adverse events (grade ≥ 3), both hematological (anemia, neutropenia, febrile neutropenia and thrombocytopenia) and non-hematological (diarrhea, hypertension, proteinuria, gastrointestinal perforation, nausea and vomiting and any thromboembolic and bleeding events).

### Analysis and presentation of results

Data were analyzed using the Review Manager 5.1.2 statistical package (Cochrane Collaboration Software) [29].

Dichotomous clinical outcomes are reported as risk ratio (RR) and survival data as hazard ratio (HR) [30]. The corresponding 95 % confidence interval (95 % CI) was calculated, considering *P* values less than 5 % (*p* < 0.05). A statistic for measuring heterogeneity was calculated through I^2^ method (25 % was considered low-level heterogeneity, 25–50 % moderate-level heterogeneity and > 50 % high-level heterogeneity) [31, 32].

To estimate the absolute gains in progression-free survival and overall survival, we calculated the meta-analytic survival curves as suggested by Parmar et al. [30]. A pooled estimate of the HR was computed by a fixed-effect model according to the inverse-variance method [33]. Thus, for effectiveness or side effects an HR or RR > 1 favors standard arm (control), whereas an HR or RR < 1 favors bevacizumab treatment.

If statistical heterogeneity was found in the meta-analysis, we performed an additional analysis using the random-effects model described by DerSimonian and Laird [34], that provides a more conservative analysis.

To assess the possibility of publication bias, we performed the funnel plot test described by Egger et al. [35]. When the pooled results were significant, the number of patients needed to treat (NNT or NNH) to cause or to prevent one event was calculated by pooling absolute risk differences in trials included in meta-analyses [36–38]. For all analyses, a forest plot was generated to display results.

In the efficacy assessment, a subgroup analysis was planned to evaluate the influence of the type of fluoropyrimidine (bolus or infusional 5-FU or capecitabine) and cytotoxic agents used (only fluoropyrimidine monotherapy, oxaliplatin-based and irinotecan-based regimens).

## Results

**Fig. 1:**
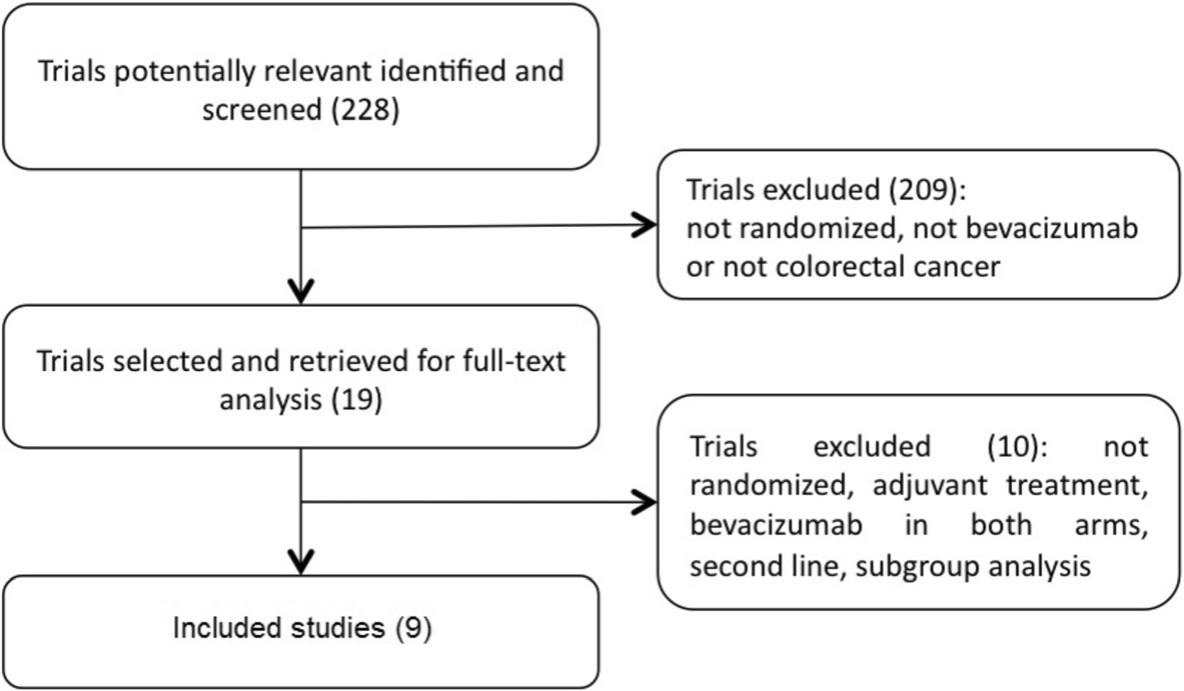
Trial selection flow

The diagram represents the flow of identification and inclusion of trials, as recommended by the Preferred Reporting Items for Systematic reviews and Meta-Analyses (PRISMA) statement [39] (Fig. 1).

In the first search, 228 references were identified and screened. Nineteen were considered of potential interest and selected for analysis in full. Of these, 10 were excluded for different reasons as described in Table 1. The final analysis included 9 trials comprising 3,914 patients (Table 2).

**Table 1:** Characteristics of excluded studies

	Reasons for exclusion
Lee 2012 [72]	Nonrandomized (cost-effectiveness analysis)
Shiroiwa 2010 [73]	Nonrandomized (cost-effectiveness analysis)
Zhang 2012 [74]	Nonrandomized
Allegra 2009/2011 [75, 76]	Adjuvant treatment
Ducreux 2009 [77]	Different comparison (bevacizumab in both arms)
Pectasides 2012 [78]	Different comparison (bevacizumab in both arms)
Souglakos 2012 [79]	Different comparison (bevacizumab in both arms)
Díaz-Rubio 2012 [80]	Different comparison (bevacizumab in both arms)
Price 2012 [81]	Subgroup analysis of another study
Moehler 2009 [82]	Nonrandomized

**Table 2:** Characteristics of randomized studies evaluating bevacizumab plus chemotherapy in patients with mCRC in first line chemotherapy

Study	*n*	Type of study	Patients	Comparison	Primary endpoint
*Regimens containing irinotecan with/without bevacizumab*					
Hurwitz 2004/2005 [14, 42] (AVF 2107)	813	Randomized, multicenter, phase III	mCRC, ECOG PS 0–1, ≥ 18 years	IFL/Bev (5 mg/kg)	OS
				IFL/placebo	
Guan 2011 [24] (ARTIST)	203	Randomized, multicenter, phase III	mCRC, ECOG PS 0–1, ≥ 18 years	IFL/Bev (5 mg/kg)	PFS and PFS rate in 6 months
				IFL	
Stathopoulos 2010 [44]	222	Randomized, phase III	mCRC, ECOG PS 0–2, ≥ 18 years	IFL/Bev (7.5 mg/kg)	OS
				IFL	
*Regimens containing oxaliplatin with/without bevacizumab*					
Saltz/Cassidy 2008/2011 [45, 46] (NO16966)	1400	Randomized, multicenter, phase III	mCRC, ECOG PS 0–1, ≥ 18 years	XELOX or FOLFOX/Bev (5 mg/kg or 7.5 mg/kg)	PFS
				XELOX or FOLFOX/placebo	
*Regimens containing oxaliplatin or irinotecan with/without bevacizumab*					
Passardi 2013/2015 [21, 23] (ITACA)^b^	370	Randomized, multicenter, phase III	mCRC, ECOG PS 0–2, ≥ 18 years	FOLFOX or FOLFIRI/Bev (5 mg/kg)	PFS
				FOLFOX or FOLFIRI	
*Regimens containing only 5-FU with/without bevacizumab*					
Kabinnavar 2003 [47]	104	Randomized, multicenter, phase III	mCRC, ECOG PS 0–1, ≥ 18 years	5-FU/LV	TTP and ORR
				5-FU/LV/Bev (5 mg/kg)	
				5-FU/LV/Bev (10 mg/kg)	
Kabinnavar 2005 [48]	209	Randomized, multicenter, phase III	mCRC, ECOG PS 1–2, ≥ 65 years	5-FU/LV/Bev (5 mg/kg)	OS
				5-FU/LV/placebo	
*Regimens containing only capecitabine with/without bevacizumab*					
Tebutt 2010 [40] (MAX)	313^a^	Randomized, multicenter, phase III	mCRC, ECOG PS 0–2, ≥ 18 years	Capecitabine/Bev (7.5 mg/kg)	PFS
				Capecitabine	
Cunningham 2013 [22] (AVEX)	280	Randomized, multicenter, phase III	mCRC, ECOG PS 0–2, ≥ 70 years	Capecitabine/Bev (7.5 mg/kg)	PFS
				Capecitabine	
Chemotherapy protocols:					
Hurwitz 2004/2005 [14, 42] - (AVF 2107)					
IFL/Placebo: 5-FU: 500 mg/m^2^, *bolus* + LV: 20 mg/m^2^, during 2 h + irinotecan: 125 mg/m^2^, once/week for 4 weeks every 6 weeks.					
IFL/Bev: same chemotherapy regimen + bevacizumab: 5 mg/kg intravenously every 15 days until progression.					
Guan 2011 [24] - (ARTIST)					
IFL/Placebo: 5-FU: 500 mg/m^2^ + LV: 20 mg/m^2^ (infusion: 6–8 h) + irinotecan: 125 mg/m^2^, once/week for 4 weeks every 6 weeks.					
IFL/Bev: same chemotherapy regimen + bevacizumab: 5 mg/kg intravenously every 15 days until progression.					
Stathopoulos 2010 [44]					
IFL: 5-FU: 500 mg/m^2^ + LV: 200 mg/m^2^ + irinotecan: 135 mg/m^2^, in Day 1 (D1) every 3 weeks.					
IFL/Bev: same chemotherapy regimen + bevacizumab: 7.5 mg/kg intravenously every 3 weeks until progression.					
Saltz/Cassidy 2008/2011 [45, 46] - (NO16966)					
FOLFOX/placebo: LV: 200 mg/m^2^/day intravenously in 2 h + 5-FU: 400 mg/m^2^/day in *bolus*, followed by 600 mg/m^2^/day in 22 h in Days 1 and 2 + oxaliplatin: 85 mg/m^2^, in 2 h, in D1;					
FOLFOX/Bev: same chemotherapy regimen + bevacizumab: 5 mg/kg, Day 1; every 15 days;					
XELOX/placebo: capecitabine: 1000 mg/m^2^ orally, twice/day, for 14 days + oxaliplatin: 130 mg/m^2^ intravenously in D1;					
XELOX/Bev: same chemotherapy regimen + bevacizumab: 7.5 mg/kg in D1; every 21 days, until progression.					
Passardi 2013/2015 [21, 23] - (ITACA)					
FOLFOX: LV: 100 mg/m^2^/day intravenously D1 and D2 + 5-FU: 400 mg/m^2^/day in *bolus* D1 and D2, followed by 600 mg/m^2^ in 22 h in Days 1 and 2 + oxaliplatin: 85 mg/m^2^, in 2 h, in D1;					
FOLFIRI: LV: 100 mg/m^2^/day intravenously D1 and D2 + 5-FU: 400 mg/m^2^/day in *bolus* D1 and D2, followed by 600 mg/m^2^ in 22 h in Days 1 and 2 + irinotecan 180 mg/m^2^, in D1;					
FOLFOX or FOLFIRI/Bev: same chemotherapy regimen + bevacizumab: 5 mg/kg, Day 1; every 15 day;					
Kabinnavar 2003 [47]					
5-FU/LV: 5-FU: 500 mg/m^2^/LV: 500 mg/m^2^, weekly for 6 weeks every 8 weeks.					
5-FU/LV/Bev (5 mg/kg): same chemotherapy regimen + bevacizumab: 5 mg/kg, Day 1, every 15 day;					
5-FU/LV/Bev (10 mg/kg): same chemotherapy regimen + bevacizumab: 10 mg/kg, Day 1, every 15 day;					
Kabinnavar 2005 [48]					
5-FU/LV: 5-FU: 500 mg/m^2^/LV: 500 mg/m^2^, weekly for 6 weeks every 8 weeks + placebo every 15 day.					
5-FU/LV/Bev (5 mg/kg): same chemotherapy regimen + bevacizumab: 5 mg/kg, Day 1, every 15 day;					
Tebutt 2010 [40] (MAX)^a^					
Capecitabine: 1000–1250 mg/m^2^ orally, twice/day, for 14 days;					
Capecitabine/Bev: same chemotherapy regimen + bevacizumab: 7.5 mg/kg D1; every 21 days, until progression.					
Cunningham 2013 [22] - (AVEX)					
Capecitabine: 1000 mg/m^2^ orally, twice/day, for 14 days;					
Capecitabine/Bev: same chemotherapy regimen + bevacizumab: 7.5 mg/kg D1; every 21 days, until progression.					

*Abbreviations*: *mCRC* metastatic colorectal cancer, *Bev* bevacizumab, *IFL* fluorouracil/leucovorin + irinotecan, *5-FU* fluorouracil, *LV* leucovorin, *OS* overall survival, *PFS* progression-free survival, *FOLFOX* bolus and infusional fluorouracil/leucovorin + oxaliplatin, *XELOX* oxaliplatin + capecitabine, *ECOG* Eastern Cooperative Oncology Group, *PS* performance status, *TTP* time to progression, *ORR* overall response rate

^a^Excluded patients with mitomycin; ^b^FOLFOX4 was used in 60 % of the patients and FOLFIRI in 40 %

A comprehensive analysis was performed regarding the presence of relevant biomarkers, such as VEGF-A isoform or *KRAS* status, which might have predicted superior efficacy for patients treated with bevacizumab or other particular regimens. Three studies reported separated data of efficacy for patients with wild type (WD) or mutated (MT) *KRAS.* Mutation status *KRAS* was determined for patients participating in the ITACA trial [21, 23], also for 315 (66,9 %) of those on MAX trial [22, 40, 41] and 230 patients (28.3 %) on the AVF2107 study [14, 42, 43].

Quality assessments for eligible trials were evaluated and performed by extracting key methodological characteristics from published trials (Additional file 1: Table S1).

### Characteristics and results of included studies

#### Studies containing chemotherapy (irinotecan-based) + bevacizumab

Bevacizumab was associated with irinotecan in 3 randomized studies [14, 24, 42, 44].

#### AVF 2107 trial

This multicenter, placebo-controlled study [14] analyzed patients with mCRC and measurable disease. Patients were initially randomized 1:1:1 to 3 groups: placebo combined with chemotherapy (IFL regimen: irinotecan + fluorouracil + leucovorin); bevacizumab (5 mg/kg) every 15 days combined with chemotherapy (IFL regimen); and bevacizumab combined with 5-FU and leucovorin (abandoned after the safety of bevacizumab + irinotecan was well established). Treatment continued until progression of disease. The primary endpoint was overall survival (Table 2). In the ITT analysis, 411 patients were randomized to the group IFL + placebo and 402 patients to the group IFL + bevacizumab.

The association of bevacizumab to the IFL regimen significantly increased the objective response rate, compared to IFL + placebo (44.8 % vs. 34.8 %; *p* = 0.004) (Table 3). Progression-free survival (10.6 months vs. 6.2 months; *p* < 0.001) and overall survival (20.3 months vs. 15.6 months; *p* < 0.001) were higher in the group receiving bevacizumab (Table 3).

**Table 3:** Efficacy results of randomized studies evaluating bevacizumab plus chemotherapy in patients with mCRC in first-line treatment

Study	*n* (ITT)	Comparison	Response rate	PFS	OS
				HR (95 % CI)	HR (95 % CI)
*Regimens containing irinotecan with/without bevacizumab*					
Hurwitz 2004/2005 [14, 42] (AVF 2107)	402	IFL/Bev	44.8 %	10.6 months	20.3 months
	411	IFL/placebo	34.8 %	6.2 months	15.6 months
			*p* = 0.004	HR: 0.54 (0.37–0.78)	HR: 0.66 (0.52–0.85)
Guan 2011 [24] (ARTIST)	139	IFL/Bev	35.3 %	8.3 months	18.7 months
	64	IFL	17.2 %	4.2 months	13.4 months
			*p* = 0.013	HR: 0.44 (0.31–0.63)	HR: 0.62 (0.41–0.95)
Stathopoulos 2010 [44]	114	IFL/Bev	36.8 %	NR	22 months
	108	IFL	35.2 %		25 months
			*p* = NS		HR: 1.05 (0.81–1.36)^b^
*Regimens containing oxaliplatin with/without bevacizumab*					
Saltz/Cassidy 2008/2011 [45, 46] (NO16966)	699	XELOX or FOLFOX/Bev	47 %	9.4 months	21.3 months
	701	XELOX or FOLFOX/placebo	49 %	8.0 months	19.9 months
			*p* = 0.31	HR: 0.83 (0.72–0.95)^a^	HR: 0.89 (0.76–1.03)
*Regimens containing oxaliplatin or irinotecan with/without bevacizumab*					
Passardi 2013/2015 [21, 23] (ITACA)^c^	176	FOLFOX or FOLFIRI/Bev	50.6 %	9.6 months	20.8 months
	194	FOLFOX or FOLFIRI	50 %	8.4 months	21.3 months
			*p* = 0.865	HR: 0.86 (0.70-1.07)	HR: 1.13 (0.89-1.43)
*Regimens containing only 5-FU with/without bevacizumab*					
Kabinnavar 2003 [47] (AVF0780)	35	5-FU/LV/Bev (5 mg/kg)	40 %	9.0 months	21.5 months
	33	5-FU/LV/Bev (10 mg/kg)	24 %	7.2 months	16.1 months
	36	5-FU/LV	17 %	5.2 months	13.8 months
			(*p* = 0.08)	HR: 0.54 (0.33–0.88)	HR: NR
Kabinnavar 2005 [48] (AVF2192)	104	5-FU/LV/Bev	26 %	9.2 months	16.6 months
	105	5-FU/LV	15.2 %	5.5 months	12.9 months
			*p* = 0.055	HR: 0.50 (0.35–0.73)	HR: 0.79 (0.56–1.10)
*Regimens containing only capecitabine with/without bevacizumab*					
Tebutt 2010 [40] (MAX)	157	Capecitabine/Bev	38.1 %	8.5 months	NR
	156	Capecitabine	30.3 %	5.7 months	
			*p* = 0.16	HR: 0.63 (0.50–0.79)	HR: 0.88 (0.68–1.13)
Cunningham 2013 [22] (AVEX)	140	Capecitabine/Bev	19 %	9.1 months	20.7 months
	140	Capecitabine	10 %	5.1 months	16.8 months
			*p* = 0.04	HR: 0.53 (0.41–0.69)	HR: 0.79 (0.57–1.09)

*Abbreviations*: *mCRC* metastatic colorectal cancer, *Bev* bevacizumab, *IFL* fluorouracil/leucovorin + irinotecan, *5-FU* fluorouracil, *LV* leucovorin, *OS* overall survival, *PFS* progression-free survival, *FOLFOX* bolus and infusional fluorouracil/leucovorin + oxaliplatin, *XELOX* oxaliplatin + capecitabine, *ITT* intent to treat, *NR* not reported, *HR* hazard ratio, *CI* confidence interval, *NS* not significant

^a^97.5 % IC; ^b^calculated by the method of Parmar; ^c^FOLFOX4 was used in 60 % of the patients and FOLFIRI in 40 %

The combination of IFL + bevacizumab was well tolerated. In general, toxicity levels ≥ 3 were higher in the group treated with bevacizumab (84.9 % vs. 74 %; *p* < 0.01). There was no significant difference in the rate of thromboembolic events, proteinuria, and bleeding or gastrointestinal perforation. Hypertension (grade ≥ 3) was more frequent in the group treated with bevacizumab (11 % vs. 2.3 %; *p* <0.01) (Table 5).

#### ARTIST trial

This prospective, multicenter study [24] assessed patients diagnosed with mCRC and measurable disease. The trial included Chinese patients and randomized (2:1) 139 patients to receive bevacizumab (5 mg/kg every 15 days) combined with chemotherapy (IFL regimen) and 64 patients to receive only IFL. The treatment continued until progression of disease. Rate of progression-free survival at 6 months and duration of progression-free survival were co-primary endpoints (Table 2).

The combination of IFL + bevacizumab significantly increased the objective response rate, compared with IFL alone (35.3 % vs. 17.2 %; *p* = 0.013) (Table 3). Progression-free survival (8.3 months vs. 4.2 months; *p* < 0.001) and overall survival (18.7 months vs. 13.4 months; *p* = 0.014) were also higher in the group treated with bevacizumab (Table 3).

Patients tolerated well the association of bevacizumab and IFL. The proportion of adverse events (grade ≥ 3) was comparable between groups (IFL: 61 % vs. IFL + bevacizumab: 69 %).

#### Stathopoulos et al. trial

This study [44] analyzed 222 patients with mCRC and measurable disease. The trial randomized 144 patients to receive bevacizumab (7.5 mg/kg) plus chemotherapy (IFL regimen) every 3 weeks and 108 patients to receive IFL alone. The primary endpoint was overall survival. Treatment continued until progression of disease (Table 2).

Response rate was similar between groups (IFL + bevacizumab: 36.8 % vs. IFL: 35.2 %; *p* = NS). Overall survival was also similar between groups (IFL + bevacizumab: IFL: 22 months vs. 25 months; *p* = 0.1391) (Table 3).

In this trial, the authors reported only the overall adverse events (without stratifying by degree). Hematologic toxicities were similar between groups (leukopenia, IFL + bevacizumab: 34.2 % vs. IFL: 36.1 %; anemia, IFL + bevacizumab: 31.6 % vs. IFL: 33.3 %; thrombocytopenia, IFL + bevacizumab: 3.5 % vs. IFL: 4.6 %). Regarding non-hematological toxicities, four adverse events were more frequent in patients treated with IFL + bevacizumab than with IFL alone (hypertension: 20.2 % vs. 0 %; proteinuria: 6.1 % vs. 0 %; bleeding: 2.6 % vs. 0 %; and gastrointestinal perforation: 0.9 % vs. 0 %). Rates of nausea, vomiting and diarrhea were similar between the groups.

### Studies containing chemotherapy (oxaliplatin-based) + bevacizumab

One randomized trial analyzed the combination of bevacizumab with oxaliplatin-based regimens in the therapy of previously untreated mCRC [45, 46].

#### NO16966 trial

This multicenter study [46] associated bevacizumab with 2 chemotherapy regimens (FOLFOX or XELOX) in patients with mCRC. In the bevacizumab + FOLFOX combination, patients received bevacizumab (5 mg/kg) on day 1 of chemotherapy every 15 days. In bevacizumab + XELOX combination, patients received bevacizumab (7.5 mg/kg) on day 1 of chemotherapy every 21 days. The trial randomized 701 patients to receive XELOX/FOLFOX + bevacizumab and 699 patients to receive XELOX/FOLFOX + placebo (Table 2).

Overall response rate was similar between groups (XELOX/FOLFOX + bevacizumab: 47 % vs. XELOX/FOLFOX + placebo: 49 %; *p* = 0.31) and progression-free survival was higher in the group treated with XELOX/FOLFOX + bevacizumab (9.4 months vs. 8.0 months; *p* = 0.0023). Overall survival was also similar between groups (XELOX/FOLFOX + bevacizumab: 21.3 months vs. XELOX/FOLFOX + placebo: 19.9 months; *p* = 0.0769) (Table 3).

Hematologic toxicities were not reported. Non-hematological toxicities (grade ≥ 3) were generally 5 % higher in patients treated with XELOX/FOLFOX + bevacizumab compared with XELOX/FOLFOX + placebo.

### Studies containing chemotherapy (irinotecan or oxaliplatin-based) + bevacizumab

#### ITACA trial

This multicenter study [21] was presented at ASCO in 2013 and published in full afterwards [23]. A total of 370 (ITT) mCRC patients were randomized to receive first-line chemotherapy (FOLFOX4 or FOLFIRI) plus bevacizumab (5 mg/kg) or chemotherapy alone. The primary endpoint was progression-free survival. FOLFOX4 regimen was used by 60 % of the patients and FOLFIRI by 40 %. Results showed no statistically significant differences in progression-free survival, overall survival and overall response rate (Table 3). Hematologic toxicities were similar between the groups. Regarding non-hematological toxicities, five adverse events were more frequently found in patients treated with chemotherapy + bevacizumab than with chemotherapy alone (hypertension: 27.8 % vs. 10.8 %; fatigue: 10.3 % vs. 3.1 %; proteinuria: 22.2 % vs. 13.4 %; bleeding: 17.0 % vs. 4.6 %; and thrombosis: 21 % vs. 12.9 %). Rates of nausea, vomiting and diarrhea were similar between the groups.

### Studies containing chemotherapy (only 5-FU) + bevacizumab

Two randomized studies investigated the use of bevacizumab with chemotherapy containing only 5-FU [47, 48].

#### AVF0780 trial

This randomized study [47] evaluated the use of bevacizumab combined with chemotherapy (“Roswell Park” scheme) in 104 patients with mCRC. Patients were randomly assigned to one of three treatment groups: 36 to receive chemotherapy alone, 35 to receive chemotherapy plus low-dose bevacizumab (5 mg/kg every 2 weeks), and 33 to receive chemotherapy plus high-dose bevacizumab (10 mg/kg every 2 weeks) (Table 2).

The group treated with bevacizumab presented better overall response rate (control arm: 17 %; low-dose arm: 40 %; high-dose arm: 24 %), longer time to disease progression (control arm: 5.2 months; low-dose arm: 9.0 months; high-dose arm: 7.2 months) and longer overall survival (control arm: 13.8 months; low-dose arm: 21.5 months; high-dose arm: 16.1 months) (Table 3). Toxicity profiles are described in Tables 4 and 5. The study did not report the degree of proteinuria, however none of the patients developed nephrotic syndrome.

**Table 4:** Results of hematological adverse events (grade > 3) of the included studies that evaluated bevacizumab plus chemotherapy in mCRC

	*n*	Anemia	Neutropenia	Febrile neutropenia	Thrombocytopenia
*Regimens containing irinotecan with/without bevacizumab*					
Hurwitz 2004/2005 [14, 42] (AVF 2107)					
IFL/Bev	393	NR	37.0 %	NR	NR
IFL/placebo	397		31.1 %		
Guan 2011 [24] (ARTIST)					
IFL/Bev	141	4 %	33 %	2 %	3 %
IFL	70	1 %	19 %	2 %	4 %
Stathopoulos 2010 [44]					
IFL/Bev	114	NR	NR	NR	NR
IFL	108				
*Regimens containing oxaliplatin with/without bevacizumab*					
Saltz/Cassidy 2008/2011 [45, 46] (NO16966)					
XELOX or FOLFOX/Bev	694	NR	NR	NR	NR
XELOX or FOLFOX/placebo	675				
*Regimens containing oxaliplatin or irinotecan with/without bevacizumab*					
Passardi 2013/2015 [21, 23] (ITACA)					
FOLFOX or FOLFIRI/Bev	176	1.1 %	39.6 %	0.6 %	2.3 %
FOLFOX or FOLFIRI	194	2.6 %	42.3 %	2.1 %	1.0 %
*Regimens containing only 5-FU with/without bevacizumab*					
Kabinnavar 2003 [47] (AVF0780)					
5-FU/LV/Bev (5 mg/kg)	35	NR	5.7 %	NR	NR
5-FU/LV/Bev (10 mg/kg)	32		3.1 %		
5-FU/LV	35		2.85 %		
Kabinnavar 2005 [48] (AVF2192)					
5-FU/LV/Bev	100	NR	5 %	NR	NR
5-FU/LV	104		7 %		
*Regimens containing only capecitabine with/without bevacizumab*					
Tebutt 2010 [40] (MAX)					
Capecitabine/Bev	157	NR	0 %	2.5 %	0 %
Capecitabine	156		1.3 %	1.9 %	0 %
Cunningham 2013 [22] (AVEX)					
Capecitabine/Bev	134	NR	1 %	NR	NR
Capecitabine	136		1 %		

*Abbreviations*: *mCRC* metastatic colorectal cancer, *Bev* bevacizumab, *IFL* fluorouracil/leucovorin + irinotecan, *FOLFOX* bolus and infusional fluorouracil/leucovorin + oxaliplatin, *XELOX* oxaliplatin + capecitabine, *NR* not reported

**Table 5:** Results of non-hematological adverse events (grade > 3) of the included studies that evaluated bevacizumab plus chemotherapy in mCRC

	*n*	Diarrhea	Hypertension	Proteinuria	Gastrointestinal perforation	Nausea/vomiting	Any thromboembolic events	Bleeding
*Regimens containing irinotecan with/without bevacizumab*								
Hurwitz 2004/2005 [14, 42] (AVF 2107)								
IFL/Bev	393	32.4 %	11.0 %	0.8 %	1.5 %	NR	19.4 %	3.1 %
IFL/placebo	397	24.7 %	2.3 %	0.8 %	0 %		16.2 %	2.5 %
Guan 2011 [24] (ARTIST)								
IFL/Bev	141	26 %	4 %	1 %	1 %	13 %	1 %	1 %
IFL	70	21 %	0 %	0 %	0 %	12 %	0 %	1 %
Stathopoulos 2010 [44]								
IFL/Bev	114	NR	NR	NR	NR	NR	NR	NR
IFL	108							
*Regimens containing oxaliplatin with/without bevacizumab*								
Saltz/Cassidy 2008/2011 [45, 46] (NO16966)								
XELOX or FOLFOX/Bev	694	NR	4 %	<1 %	<1 %	NR	10 %	2 %
XELOX or FOLFOX/placebo	675		1 %	0 %	<1 %		6 %	1 %
*Regimens containing oxaliplatin or irinotecan with/without bevacizumab*								
Passardi 2013/2015 [21, 23] (ITACA)								
FOLFOX or FOLFIRI/Bev	176	8.0 %	27.8 %	22.2 %	NR	5.2 %	21 %	17.0 %
FOLFOX or FOLFIRI	194	5.7 %	10.8 %	13.4 %		3.7 %	12.9 %	4.6 %
*Regimens containing only 5-FU with/without bevacizumab*								
Kabinnavar 2003 [47] (AVF0780)								
5-FU/LV/Bev (5 mg/kg)	35	28.6 %	8.6 %	NR	NR	NR	14.3 %	0 %
5-FU/LV/Bev (10 mg/kg)	32	31.2 %	25 %				6.2 %	9.3 %
5-FU/LV	35	37.1 %	0 %				2.8 %	0%^a^
Kabinnavar 2005 [48] (AVF2192)								
5-FU/LV/Bev	100	39 %	16 %	1 %	2 %	NR	18 %	5 %
5-FU/LV	104	40 %	3 %	0 %	0 %		18 %	3 %
*Regimens containing only capecitabine with/without bevacizumab*								
Tebutt 2010 [40] (MAX)								
Capecitabine/Bev	157	17 %	3.8 %	3.2 %	1.9 %	10.2 %	12.1 %	1.3 %
Capecitabine	156	11 %	0.6 %	0.6 %	0.6 %	10.9 %	7.1 %	2.6 %
Cunningham 2013 [22] (AVEX)								
Capecitabine/Bev	134	7 %	2 %	1 %	0 %	3 %	11 %	0 %
Capecitabine	136	6 %	1 %	0 %	0 %	1 %	5 %	1 %

*Abbreviations*: *mCRC* metastatic colorectal cancer, *Bev* bevacizumab, *IFL* fluorouracil/leucovorin + irinotecan, *FOLFOX* bolus and infusional fluorouracil/leucovorin + oxaliplatin, *XELOX* oxaliplatin + capecitabine, *NR*: not reported

^a^Epistaxis and bleeding were put together

#### AVF2192 trial

This study [48] randomized patients to receive chemotherapy (“Roswell Park” scheme) plus placebo (*n* = 105) or chemotherapy plus bevacizumab (*n* = 104). The primary endpoint was overall survival. The trial included patients (aged ≥65 years) who were not optimal candidates for treatment with irinotecan [41] (Table 2).

The addition of bevacizumab to chemotherapy increased the overall response rate (5-FU + leucovorin + bevacizumab: 26.0 % vs. 5-FU + leucovorin + placebo: 15.2 %; *p* = 0.055) and progression-free survival (5-FU + leucovorin + bevacizumab: 9.2 months vs. 5-FU + leucovorin + placebo: 5.5 months; *p* = 0.0002), but had no difference in overall survival (5-FU + leucovorin + bevacizumab: 16.6 months vs. 5-FU + leucovorin + placebo: 12.9 months; *p* = 0:16) (Table 3).

Regarding adverse events, grade 3 hypertension was more frequent in the bevacizumab arm (16 % vs*.* 3 %), (Table 5).

### Studies containing chemotherapy (capecitabine only) + bevacizumab

Two randomized studies assessed bevacizumab plus chemotherapy compared to capecitabine alone [22, 40].

#### MAX trial

This multicenter phase III trial evaluated bevacizumab plus chemotherapy versus capecitabine alone [40]. The trial analyzed patients with mCRC without prior chemotherapy. Overall, 471 patients were randomly assigned to receive capecitabine; capecitabine plus bevacizumab (7.5 mg/kg); or capecitabine plus bevacizumab and mitomycin. The primary endpoint was progression-free survival. Since the chemotherapy regimen including mitomycin is not considered standard for mCRC according to the main international guidelines, that particular group was not evaluated in this meta-analysis.

Overall response rate (capecitabine + bevacizumab: 56 % vs. 43 %; *p* = 0.16) and overall survival were similar between groups (Table 3). Progression-free survival was higher in the group treated with capecitabine + bevacizumab (8.5 months vs. 5.7 months; *p* < 0.001).

Hand-foot syndrome (also known as palmar-plantar erythrodysesthesia) and diarrhea were the most common grades ≥3 toxicities (Table 5).

#### AVEX trial

This phase III trial [22] included patients (aged ≥ 70 years) with previously untreated mCRC, who were not fit candidates for oxaliplatin-based or irinotecan-based chemotherapy regimens. Patients were randomly assigned to receive bevacizumab plus capecitabine (*n* = 140) or capecitabine alone (*n* = 140). The primary endpoint was progression-free survival.

Chemotherapy plus bevacizumab achieved higher overall response rate (19 % vs. 10 %; p = 0.04) and progression-free survival (9.1 months vs. 5.1 months; *p* < 0.0001). Median overall survival was 20.7 months in the combination arm and 16.8 months in the capecitabine alone group (*p* = 0.18) (Table 3).

The frequencies of grade ≥3 adverse events related to chemotherapy, with the exception of hand-foot syndrome, remained similar between the groups as seen on Tables 4 and 5.

### Meta-analyses

The meta-analyses performed found that the combination of bevacizumab with chemotherapy resulted in higher overall response rate, progression-free survival and overall survival.

**Fig. 2:**
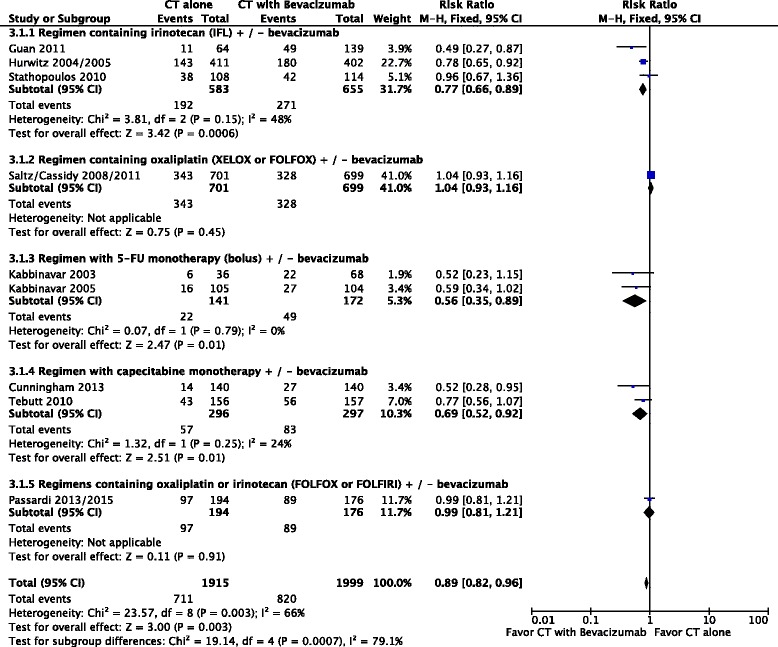
Comparative effect in objective response rates of chemotherapy with bevacizumab versus chemotherapy alone. *Abbreviations*: CT, chemotherapy

Overall response rate was higher in patients who received chemotherapy plus bevacizumab (RR = 0.89; 95 % CI: 0.82 to 0.96; 0.003; NNT = 20). Nevertheless, the results had significant heterogeneity (Chi^2^ = 23.57; df = 8 [*p* = 0.003]; I^2^ = 66 %), (Fig. 2). We performed a random-effects model analysis to better explore this heterogeneity. In this analysis, this result remained favorable to the use of chemotherapy plus bevacizumab (RR = 0.81; 95 % CI: 0.68 to 0.95; *p* = 0.01).

**Fig. 3:**
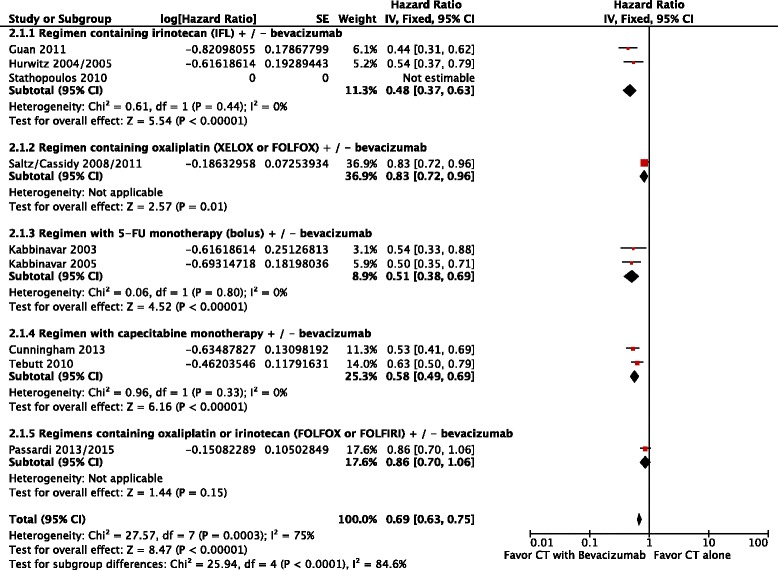
Comparative effect in progression-free survival of chemotherapy with bevacizumab versus chemotherapy alone (Fixed-effect model analysis). *Abbreviations*: CT, chemotherapy; CI, confidence interval

The progression-free survival was also higher in patients treated with chemotherapy plus bevacizumab (HR = 0.69; 95 % CI: 0.63 to 0.75; *p* < 0.00001; NNT = 3), again with significant heterogeneity (Chi^2^ = 27.5; df = 7 [*p* = 0.0003]; I^2^ = 75 %) and difference among the groups (*p* < 0.0001; I^2^ = 84.6 %), (Fig. 3). In this case, we also performed a random-effects model analysis, in which results remained favorable to the use of chemotherapy plus bevacizumab (HR = 0.61; 95 % CI: 0.51 to 0.74; *p* < 0.00001).

**Fig. 4:**
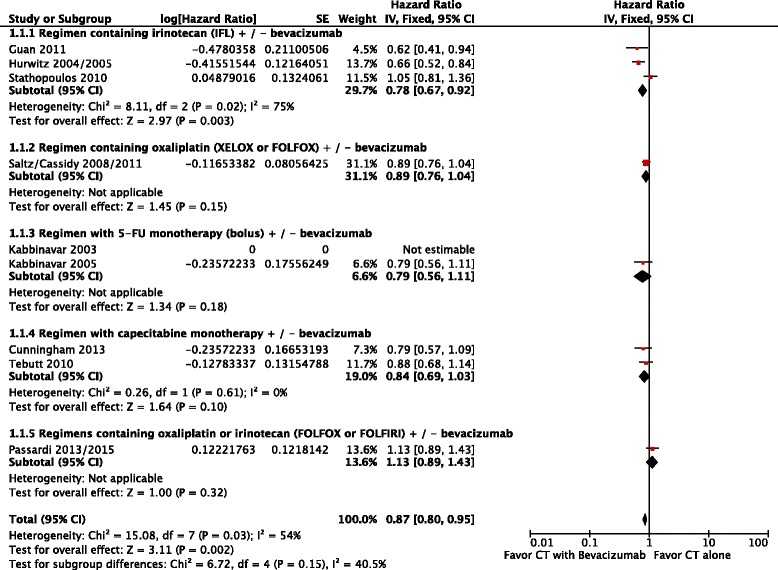
Comparative effect in overall survival of chemotherapy with bevacizumab versus chemotherapy alone (Fixed-effect model analysis). *Abbreviations*: CT, chemotherapy; CI, confidence interval

**Fig. 5:**
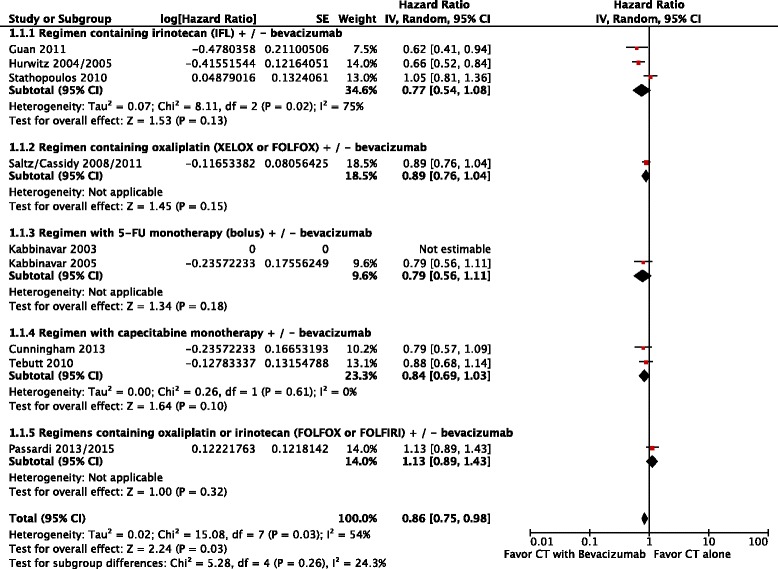
Comparative effect in overall survival of chemotherapy with bevacizumab versus chemotherapy alone (random-effects model analysis). *Abbreviations*: CT, chemotherapy; CI, confidence interval

Lastly, overall survival was higher in patients who received chemotherapy plus bevacizumab (HR = 0.87; 95 % CI: 0.80 to 0.95; p = 0.002; NNT = 7). This result had moderate heterogeneity (Chi^2^ = 15.08; df = 7 [*p* = 0.03]; I^2^ = 54 %), (Fig. 4). Analysis by random-effects model found that differences in these endpoints remained in favor of chemotherapy plus bevacizumab (HR = 0.86; 95 % CI: 0.75 to 0.98; *p* = 0.03) (Fig. 5).

As an additional attempt of exploring the heterogeneity found in survival analyses, we discarded two of the studies that included patients with mean age over 70 years [22, 48], however the results still showed significant heterogeneity. The same strategy was employed in regards to the Eastern Cooperative Oncology Group performance status (ECOG PS). When the study [48] including only patients with ECOG PS 1–2 was taken out of the analysis, heterogeneity remained present in the survival outcomes results.

Regarding adverse events and severe toxicities (grade ≥3), the group receiving chemotherapy plus bevacizumab had higher rates of hypertension (RR = 3.56 95 % CI: 2.58 to 4.92; *p* < 0.00001; NNH = 17), proteinuria (RR = 1.89; 95 % CI: 1.26 to 2.84; p = 0.002; NNH = 100), gastrointestinal perforation (RR = 3.63; 95 % CI: 1.31 to 10.09; p = 0.01; NNH = 100), any thromboembolic events (RR = 1.44; 95 % CI: 1.20 to 1.73; *p* = 0.0001; NNH = 25), and bleeding (RR = 1.81; 95 % CI: 1.22 to 2.67; *p* = 0.003; NNH = 50), without heterogeneity (Additional file 2 and 3: Figure S1 and S2).

According to the funnel plot analysis [35], the possibility of publication bias was low for all efficacy endpoints (Additional file 4).

### Subgroup analysis

#### Type of fluoropyrimidine administration

In the assessment of efficacy, a subgroup analysis evaluating type of administration demonstrated that the overall response rate was higher in patients treated with bolus fluoropyrimidine plus bevacizumab (RR = 0.74; 95 % CI: 0.64 to 0.85; *p* < 0.0001; NNT = 10) with moderate heterogeneity (Chi^2^ = 5.74; df = 4 [*p* = 0.22]; I^2^ = 30 %). Other subgroups treated with infusional chemotherapy or capecitabine-based regimens, both combined with bevacizumab, showed no statistically significant difference (infusional 5-FU: RR = 0.96; 95 % CI: 0.84 to 1:08; *p* = 0.47; and capecitabine-based: RR = 0.92; 95 % CI: 0.80 to 1.06; *p* = 0.25).

We performed a random-effects model analysis to better explore this heterogeneity. In this analysis, results remained favorable to the use of chemotherapy with bolus fluoropyrimidine plus bevacizumab (RR = 0.73; 95 % CI: 0.59 to 0.90; *p* = 0.004).

The progression-free survival was also higher in patients receiving bolus 5-FU plus bevacizumab (HR = 0.50; 95 % CI: 0.41 to 0.60; *p* < 0.00001; NNT = 2), with no heterogeneity (Chi^2^ = 0.76; df = 3 [p = 0.86]; I^2^ = 0 %) and also capecitabine-based regimens plus bevacizumab (HR = 0.66; 95 % CI: 0.58 to 0.75; *p* < 0.00001; NNT = 3), although with significant heterogeneity (Chi^2^ = 5.22; df = 2 [p = 0.07]; I^2^ = 62 %). There was no significant difference in progression-free survival with infusional 5-FU plus bevacizumab (HR = 0.88; 95 % CI: 0.76 to 1.01; *p* = 0.07).

In this instance, we also performed a random-effects model analysis, in which results remained favorable in the groups treated with bolus 5-FU plus bevacizumab (HR = 0.50; 95 % CI: 0.41 to 0.60; *p* < 0.00001) or with capecitabine-based regimens plus bevacizumab (HR = 0.64; 95 % CI: 0.52 to 0.80; *p* < 0.0001).

Overall survival had similar results to those seen for progression-free survival, with statistically significant difference for bolus 5-FU plus bevacizumab (HR = 0.78; 95 % CI: 0.68 to 0.91; *p* = 0.001; NNT = 4) with significant heterogeneity (Chi^2^ = 8.11; df = 3 [*p* = 0.04]; I^2^ = 63 %) and capecitabine-based regimens plus bevacizumab (HR = 0.84; 95 % CI: 0.73 to 0.97; *p* = 0.02; NNT = 6) without heterogeneity (Chi^2^ = 0.26; df = 2 [*p* = 0.88]; I^2^ = 0 %). There was no significant difference in overall survival with infusional 5-FU plus bevacizumab (HR = 1.03; 95 % CI: 0.87 to 1.21; p = 0.76).

Once more, the random-effects model analysis showed that results remained favorable to the use of bolus 5-FU plus bevacizumab (HR = 0.77; 95 % CI: 0.60 to 0.99; *p* = 0.05) or capecitabine-based regimens plus bevacizumab (HR = 0.84; 95 % CI: 0.73 to 0.97; *p* = 0.02).

#### Type of cytotoxic agents

According to the type of systemic therapy, the overall response rate was higher in the subgroup receiving a regimen containing irinotecan (IFL or FOLFIRI) plus bevacizumab (RR = 0.82; 95 % CI: 0.71 to 0.94; *p* = 0.004; NNT = 13), but with moderate heterogeneity (Chi^2^ = 8.33; df = 3 [*p* = 0.04]; I^2^ = 64 %) and monotherapy with fluoropyrimidine plus bevacizumab (RR = 0.65; 95 % CI: 0.51 to 0.83; p = 0.0005; NNT = 10), with no heterogeneity (Chi^2^ = 2.06; df = 3 [p = 0.56]; I^2^ = 0 %). In subgroups receiving oxaliplatin-containing regimens (XELOX or FOLFOX) plus bevacizumab response rates were similar to those seen in the patients treated without bevacizumab (RR = 1.02; 95 % CI: 0.92 to 1.13; p = 0.72) with moderate heterogeneity (Chi^2^ = 1.35; df = 1 [*p* = 0.24]; I^2^ = 26 %).

We performed a random-effects model analysis to better explore this heterogeneity. In this analysis, this result remained favorable to the use of monotherapy with fluoropyrimidine plus bevacizumab (RR = 0.66; 95 % CI: 0.52 to 0.84; p = 0.0009).

Progression-free survival was higher in subgroups treated with irinotecan-containing regimens (IFL or FOLFIRI) plus bevacizumab (HR = 0.57; 95 % CI: 0.47 to 0.70; *p* < 0.00001; NNT = 2), with heterogeneity (Chi^2^ = 4.86; df = 2 [*p* = 0.09]; I^2^ = 59 %). The same was seen in patients treated with oxaliplatin-containing regimen (XELOX or FOLFOX) plus bevacizumab (HR = 0.86; 95 % CI: 0.76 to 0.98; *p* = 0.02; NNT = 7), with moderate heterogeneity (Chi^2^ = 1.29; df = 1 [p = 0.26]; I^2^ = 23 %), and also in those receiving fluoropyrimidine monotherapy plus bevacizumab (HR = 0.56; 95 % CI: 0.49 to 0.65; *p* < 0.00001; NNT = 2), without heterogeneity (Chi^2^ = 1.57; df = 3 [*p* = 0.67]; I^2^ = 0 %).

In a random-effects model analysis results remained favorable to the use of irinotecan-containing chemotherapy (IFL or FOLFIRI) plus bevacizumab (HR = 0.57; 95 % CI: 0.41 to 0.78; *p* = 0.0004) and fluoropyrimidine monotherapy plus bevacizumab.

Regarding overall survival results, it was not possible to include the ITACA trial [21, 23], since data by treatment regimens (FOLFOX or FOLFIRI) were not reported.

Overall survival was higher for irinotecan-containing regimens (IFL) plus bevacizumab (HR = 0.78; 95 % CI: 0.67 to 0.92; p = 0.003; NNT = 4), although with significant heterogeneity (Chi^2^ = 8.11; df = 2 [*p* = 0.02]; I^2^ = 75 %), and for fluoropyrimidine monotherapy plus bevacizumab (HR = 0.83; 95 % CI: 0.70 to 0.99; *p* = 0.04; NNT = 6), without heterogeneity (Chi^2^ = 0.36; df = 2 [*p* = 0.83]; I^2^ = 0 %).

### *KRAS* status

We also explored if there were any interaction between the *KRAS* status and the effect of treatment. In the subgroup analysis of patients who received chemotherapy plus bevacizumab we did not observed any difference in response rates between patients with wild type or mutated *KRAS* (WD: RR = 0.80; 95 % CI: 0.61 to 1.05; *p* = 0.11 and MT: RR = 0.99; 95 % CI: 0.65 to 1.51; *p* = 0.97).

The interaction test was negative for subgroup differences. The progression-free survival was higher in patients treated with chemotherapy plus bevacizumab regardless of *KRAS* status (WD: HR = 0.71; 95 % CI: 0.60 to 0.85; *p* = 0.0002 and MT: HR = 0.70; 95 % CI: 0.54 to 0.90; *p* = 0.006). The interaction test was negative for subgroup differences. Overall survival was similar between patients with wild type or mutated *KRAS* (WD: HR = 0.79, CI95% = 0.58 to 1.07; *p* = 0.13 and MT: HR = 0.91, CI95% = 0.63 to 1.32; p = 0.62). The interaction test was negative for subgroup differences.

## Discussion

Several guidelines around the world consider the combination of bevacizumab plus chemotherapy with 5-FU plus oxaliplatin or irinotecan (FOLFOX, FOLFIRI, IFL, XELOX, etc.) as an option for first-line treatment of mCRC, especially in patients with mutated KRAS [1, 4, 49–60]. We found 9 systematic reviews with meta-analysis evaluating the use of bevacizumab combined with chemotherapy in the treatment of mCRC [16–20, 61–64].

The first review, published in 2009 [19], grouped patients treated with standard chemotherapy + bevacizumab. This review included 5 studies [14, 46–48, 65], 4 in first-line and 1 in second-line treatment. Subgroup analysis performed with patients treated with chemotherapy + bevacizumab in first-line demonstrated a progression-free survival in favor of the group receiving bevacizumab (HR: 0.61; 95 % CI: 0.45 to 0.83; *p* = 0.0017). There was also a difference in overall survival favoring the group treated with bevacizumab (HR: 0.81; 95 % CI: 0.73 to 0.90; p = 0.00009).

Later, in 2010, another systematic review [16] corroborated the results of the previous publication including the same 5 studies that evaluated standard chemotherapy + bevacizumab [14, 46–48, 65]. Generally, progression-free survival (HR = 0.63; 95 % CI: 0.49 to 0.81; *p* = 0.0004) and overall survival (HR = 0.79; 95 % CI: 0.69 to 0.90; *p* = 0.0005) favored the group treated with bevacizumab.

In 2011, a new systematic review [17] included - in addition to the 5 studies [14, 46–48, 65] described in previous reviews [16, 19] - the results of another randomized trial evaluating chemotherapy + bevacizumab in mCRC first-line treatment [40]. Both progression-free survival (HR = 0.62; 95 % CI: 0.52 to 0.74; *p* < 0.00001) and overall survival (HR = 0.80; 95 % CI: 0.71 to 0.91; *p* = 0.0004) favored chemotherapy with bevacizumab.

In 2012, Macedo et al. [18] conducted a systematic review with meta-analysis focusing on the subgroups of chemotherapies used in trials with bevacizumab. In this review, 6 studies compared standard chemotherapy alone versus chemotherapy plus bevacizumab in patients with mCRC without prior treatment. This meta-analysis concluded that bevacizumab was an effective agent for mCRC first-line treatment. However, its effectiveness was observed in specific types of chemotherapy such as bolus fluorouracil, capecitabine-based regimens, and irinotecan containing schemes.

In 2013, Lv et al. [61] conducted a systematic review with meta-analysis of 10 randomized trials including most of the options of chemotherapy considered standard for mCRC. The overall analysis included patients in first-line and second-line treatment for mCRC and also those in adjuvant therapy. The overall result favored the arms treated with bevacizumab regarding progression-free survival (HR = 0.59; 95 % CI: 0.51 to 0.67) and overall survival (HR = 0.78; 95 % CI: 0.70 to 0.87).

All systematic reviews with meta-analysis published until 2013 presented progression-free survival and overall survival results that favored the chemotherapy plus bevacizumab combination, despite heterogeneity among subgroups.

However, in 2014, other publication [20] showed that the addition of bevacizumab to first-line chemotherapy did not add clinical benefit for overall survival. This meta-analysis included 7 studies and raised questions about the real benefit of chemotherapy plus bevacizumab combination in first-line therapy.

Three meta-analyses evaluating the impact of the addition of bevacizumab to chemotherapy in patients with CRC were published in 2015 [62–64].

The first, by Zhang et al. [64] included 9 studies, either randomized-controlled trials or cohorts that assessed bevacizumab plus standard chemotherapy in patients with mCRC in first-line treatment. The author did not perform subgroup analyses by mode of administration of type of cytotoxic drug in combination with bevacizumab. In general, results were favorable to the addition of bevacizumab regarding overall response rate (OR = 1.57, 95 % CI: 1.17 to 2.11, p = 0.003), progression-free survival (HR = 0.56, 95 % CI: 0.46 to 0.69, *p* < 0.00001) and overall survival (HR = 0.83, 95 % CI: 0.76 to 0.91, *p* < 0.0001).

The second meta-analysis, by CY, et al. [63], included 10 studies on the use of bevacizumab in patients with CRC. Similar to Lv et al. [61], the overall analysis included patients in first-line and second-line treatment for mCRC and also those in adjuvant therapy. Moreover, there were no subgroup analyses regarding the impact of treatment in each line of therapy or type of cytotoxic drug. The overall result favored the arms treated with bevacizumab regarding progression-free survival (HR = 0.61; 95 % CI: 0.53 to 0.71) and overall survival (HR = 0.84; 95 % CI: 0.74 to 0.96).

The third meta-analysis, by Hu et al. [62], included 7 trials on the addition of bevacizumab to standard chemotherapy for patients with mCRC in first-line of treatment. Overall response rates were higher for those treated with bevacizumab (RR = 1.17, 95 % CI: 1.06 to 1.28, *p* = 0.001), as were progression-free survival (HR = 0.67, 95 % CI: 0.61 to 0.72) and overall survival (HR = 0.67, 95 % CI: 0.61 to 0.72).

Our review included the results of 3 studies [21–24] that also evaluated the addition of bevacizumab to first-line treatment of mCRC, which were not included in any the previously published meta-analyses. Our results demonstrated that the overall response rate, progression-free survival and overall survival were higher in patients who received the combination of chemotherapy plus bevacizumab in a fixed effects model analysis, but with heterogeneity. These results remain favorable to the same combination with bevacizumab, even after a random-effects model analysis was performed.

One hypothesis that may explain the heterogeneity found in this and other systematic reviews is the difference in bevacizumab doses and individual inclusion criteria – as age and ECOG PS of patients – for each study.

The wide range regarding mean age of patients detected on the included trials might have contributed to the heterogeneity seen in all meta-analyses performed to this date. Two trials included patients with mean age between 50 and 60 years [14, 24, 42]; four had patients with mean age between 60 and 70 years [21, 23, 40, 44–46] and two included patients with mean age over 70 years [22, 48].

Levels of ECOG performance status of included patients were also distinct along the trials, varying from PS 0–1 [14, 24, 42, 45–47]; PS 0–2 [21–23, 40, 44] and PS 1–2 [48].

The mode of chemotherapy administration and type of cytotoxic drug also seemed to influence the results, since in the subgroups analysis the response rate was higher in patients who received bolus fluoropyrimidine plus bevacizumab and in those treated with irinotecan-containing regimens or fluoropyrimidine monotherapy plus bevacizumab.

Progression-free survival and the overall survival were also influenced by the same variables, as better results were seen in patients receiving bolus 5-FU or capecitabine-based chemotherapy plus bevacizumab. Regarding the type of cytotoxic regimen, all subgroups (irinotecan-containing, oxaliplatin-containing and fluoropyrimidine monotherapy) had favorable results on progression-free survival with the addition of bevacizumab. However, only patients treated with irinotecan-containing regimens (IFL) or fluoropyrimidine monotherapy had statistically significant results in overall survival with the association of bevacizumab.

Oxaliplatin might not be an ideal partner for bevacizumab, as pointed out by Macedo et al. [18]. Both studies that combined oxaliplatin-based chemotherapy [21, 23, 45, 46] plus bevacizumab failed to show benefit in overall survival. In the trial by Passardi [21, 23] FOLFOX4 was used in 60 % of the patients. Separate data on overall survival FOLFIRI or FOLFOX were not reported, however. The bevacizumab dose in studies using oxaliplatin-based chemotherapy was similar to those seen in studies with irinotecan regimens. Also, the ECOG PS of patients was comparable to that seen in irinotecan-based or 5FU monotherapy regimens.

The efficacy results of our meta-analysis were similar to those found by Macedo et al. [18], who also assessed the addition of bevacizumab in different settings, albeit with fewer studies included. To our knowledge, ours is the only meta-analysis that evaluated subgroups of patients by type of cytotoxic treatment and mode of administration.

This systematic review is comprised by trials published through a span of more than 10 years. This opens issues regarding the appropriateness of pooling the results of older trials from the standpoint of current standards of care in advanced colorectal cancer. Sensitivity analyses were performed excluding AVF 2107 [14, 42] and Kabinnavar et al. [47, 48] trials, both with patient accrual taking place in the early 2000s (data not shown). Although overall survival data didn’t statistically favor bevacizumab-containing regimens (HR = 0.92; 95 % CI 0.83, 1.01), benefits in PFS persisted in the same magnitude as observed in the main analysis (HR = 0.72; 95 % CI 0.66, 0.79). It should be noted that this exploratory analysis carries some potential biases: Most trials included were conducted with sample calculation taking into account that the primary endpoint was PFS, limiting the chance for identification of an overall survival benefit even if this was a true effect of bevacizumab containing regimens. Furthermore, crossover to bevacizumab was not allowed in some trials [14, 42, 48] but it did occur in others [22, 46, 47], while some studies did not explicitly report such information [24, 40, 44]. Nevertheless, PFS lengthening have been shown to strongly correlate with improvements in overall survival in advanced colorectal cancer [66, 67].

Since bevacizumab is an antibody against VEGF, another aspect that needs further clarification is the potential benefit of VEGF-A isoform plasma levels in patients who are eligible for this targeted therapy. It is known that VEGF is overexpressed in various human malignancies [68] and it is considered to be an important regulator of physiologic and pathologic angiogenesis [44, 69]. In many instances, VEGF is correlated with an adverse prognosis (increased risk of tumor recurrence and metastasis and decreased survival) [48]. Recently, a systematic review with meta-analysis presented at the ESMO Meeting [70] explored this correlation and, despite the small number of included studies, showed that VEGF-A plasma levels seemed to predict benefit with bevacizumab in early and advanced breast cancer. Appraisal of VEGF-A levels in patients with mCRC might similarly help to select those with the greatest potential for treatment response.

None of the meta-analyses evaluating the addition of bevacizumab to standard chemotherapy in patients with mCRC was able to identify the potential benefit of VEGF-A isoform plasma levels, or other efficacy biomarkers, since the included studies did not report on that outcome. KRAS gene mutation status was not predictive of bevacizumab outcome in patients with mCRC. Efficacy results of chemotherapy alone versus chemotherapy plus bevacizumab did not differ in regard to biomarker status.

Regarding adverse events and severe toxicities (grade ≥ 3), the group receiving chemotherapy plus bevacizumab had higher rates of hypertension, proteinuria, gastrointestinal perforation and any thromboembolic events.

This profile of toxicity warrants a greater level of attention to those patients at an increased risk for thromboembolic events and gastrointestinal perforation (such as the elderly or debilitated patients with Speritoneal carcinomatosis). As seen in another systematic review with meta-analysis evaluating the use of bevacizumab plus chemotherapy in lung cancer [71], hypertension and proteinuria are usually controllable events and do not require permanent discontinuation of therapy.

## Conclusion

The combination of chemotherapy plus bevacizumab increased the response rate, progression-free survival and overall survival of previously untreated patients diagnosed with mCRC.

Regarding the mode of fluoropyrimidine administration, both bolus (IFL) and capecitabine-based regimens combined with bevacizumab presented better results in survival outcomes. As for the type of systemic therapy associated with bevacizumab, regimens containing irinotecan and therapy with fluoropyrimidine monotherapy showed better efficacy results. Thus, patients who are not candidates for oxaliplatin-based or irinotecan-based chemotherapy regimens may benefit from the treatment with bevacizumab plus fluoropyrimidine monotherapy regimen.

## Additional files

Additional file 1: Quality assessment (risk of bias) of randomized studies evaluating bevacizumab plus chemotherapy in patients with mCRC in first line chemotherapy. (DOCX 46 kb) Additional file 2: Figure S1. Comparative effect in hematologic toxicities of chemotherapy with bevacizumab versus chemotherapy alone. (PDF 503 kb) Additional file 3: Figure S2. Comparative effect in non-hematologic toxicities of chemotherapy with bevacizumab versus chemotherapy alone. (PDF 864 kb) Additional file 4: Assessment of publication bias. Funnel plot for objective response rates, progression-free survival and overall survival in this meta-analysis. (DOCX 352 kb)

## References

[1] National Comprehensive Cancer Network (NCCN) Clinical Practice Guidelines in Oncology: Colon Cancer (Version 2.2016). http://www.nccn.org/professionals/physician_gls/pdf/colon.pdf 10.6004/jnccn.2016.0051 27059193

[2] Siegel RL, Miller KD, Jemal A (2015). Cancer statistics, 2015. CA Cancer J Clin.

[3] Parkin DM, Pisani P, Ferlay J (1999). Global cancer statistics. CA Cancer J Clin.

[4] Van Cutsem E, Oliveira J (2009). Advanced colorectal cancer: ESMO clinical recommendations for diagnosis, treatment and follow-up. Ann Oncol.

[5] Midgley R, Kerr D (1999). Colorectal cancer. Lancet (London, England).

[6] Jackson NA, Barrueco J, Soufi-Mahjoubi R, Marshall J, Mitchell E, Zhang X (2009). Comparing safety and efficacy of first-line irinotecan/fluoropyrimidine combinations in elderly versus nonelderly patients with metastatic colorectal cancer: findings from the bolus, infusional, or capecitabine with camptostar-celecoxib study. Cancer.

[7] Colucci G, Gebbia V, Paoletti G, Giuliani F, Caruso M, Gebbia N (2005). Phase III randomized trial of FOLFIRI versus FOLFOX4 in the treatment of advanced colorectal cancer: a multicenter study of the Gruppo Oncologico Dell’Italia Meridionale. J Clin Oncol.

[8] Schmoll HJ, Cartwright T, Tabernero J, Nowacki MP, Figer A, Maroun J (2007). Phase III trial of capecitabine plus oxaliplatin as adjuvant therapy for stage III colon cancer: a planned safety analysis in 1,864 patients. J Clin Oncol.

[9] Fuchs CS, Marshall J, Mitchell E, Wierzbicki R, Ganju V, Jeffery M (2007). Randomized, controlled trial of irinotecan plus infusional, bolus, or oral fluoropyrimidines in first-line treatment of metastatic colorectal cancer: results from the BICC-C Study. J Clin Oncol.

[10] Van Cutsem E, Twelves C, Cassidy J, Allman D, Bajetta E, Boyer M (2001). Oral capecitabine compared with intravenous fluorouracil plus leucovorin in patients with metastatic colorectal cancer: results of a large phase III study. J Clin Oncol.

[11] de Gramont A, Figer A, Seymour M, Homerin M, Hmissi A, Cassidy J (2000). Leucovorin and fluorouracil with or without oxaliplatin as first-line treatment in advanced colorectal cancer. J Clin Oncol Off J Am Soc Clin Oncol.

[12] Douillard JY, Cunningham D, Roth AD, Navarro M, James RD, Karasek P (2000). Irinotecan combined with fluorouracil compared with fluorouracil alone as first-line treatment for metastatic colorectal cancer: a multicentre randomised trial. Lancet (London, England).

[13] Saltz LB, Cox JV, Blanke C, Rosen LS, Fehrenbacher L, Moore MJ (2000). Irinotecan plus fluorouracil and leucovorin for metastatic colorectal cancer. Irinotecan Study Group. N Engl J Med.

[14] Hurwitz H, Fehrenbacher L, Novotny W, Cartwright T, Hainsworth J, Heim W (2004). Bevacizumab plus irinotecan, fluorouracil, and leucovorin for metastatic colorectal cancer. N Engl J Med.

[15] US Food and Drug Adminisration (FDA). http://www.fda.gov/drugs/drugsafety/postmarketdrugsafetyinformationforpatientsandproviders/ucm193900.htm. Accessed Jan 2014.

[16] Welch S, Spithoff K, Rumble RB, Maroun J (2010). Bevacizumab combined with chemotherapy for patients with advanced colorectal cancer: a systematic review. Ann Oncol.

[17] Galfrascoli E, Piva S, Cinquini M, Rossi A, La Verde N, Bramati A (2011). Risk/benefit profile of bevacizumab in metastatic colon cancer: a systematic review and meta-analysis. Dig Liver Dis.

[18] Macedo LT, da Costa Lima AB, Sasse AD (2012). Addition of bevacizumab to first-line chemotherapy in advanced colorectal cancer: a systematic review and meta-analysis, with emphasis on chemotherapy subgroups. BMC Cancer.

[19] Wagner AD, Arnold D, Grothey AA, Haerting J, Unverzagt S (2009). Anti-angiogenic therapies for metastatic colorectal cancer. Cochrane Database Syst Rev.

[20] Chen YX, Yang Q, Kuang JJ, Chen SY, Wei Y, Jiang ZM (2014). Efficacy of adding bevacizumab in the first-line chemotherapy of metastatic colorectal cancer: evidence from seven randomized clinical trials. Gastroenterol Res Pract.

[21] Passardi A, Scarpi E, Cavanna L, Fontana A, Vertogen B, Ruscelli S (2013). Effectiveness of bevacizumab added to gold standard chemotherapy in metastatic colorectal cancer (mCRC): Final results from the Itaca randomized clinical trial. J Clin Oncol.

[22] Cunningham D, Lang I, Marcuello E, Lorusso V, Ocvirk J, Shin DB (2013). Bevacizumab plus capecitabine versus capecitabine alone in elderly patients with previously untreated metastatic colorectal cancer (AVEX): an open-label, randomised phase 3 trial. Lancet Oncol.

[23] Passardi A, Nanni O, Tassinari D, Turci D, Cavanna L, Fontana A (2015). Effectiveness of bevacizumab added to standard chemotherapy in metastatic colorectal cancer: final results for first-line treatment from the ITACa randomized clinical trial. Ann Oncol.

[24] Guan ZZ, Xu JM, Luo RC, Feng FY, Wang LW, Shen L (2011). Efficacy and safety of bevacizumab plus chemotherapy in Chinese patients with metastatic colorectal cancer: a randomized phase III ARTIST trial. Chin J Cancer.

[25] Dickersin K, Scherer R, Lefebvre C (1994). Identifying relevant studies for systematic reviews. BMJ.

[26] Clarke M, Oxman AD, (Editors). Cochrane Reviewers Handbook 4.1.1 [updated December 2000] In: The Cochrane Library, Issue 4, 2000. Oxford: Update Software; 2000.

[27] Castro AA, Clark OA, Atallah AN (1999). Optimal search strategy for clinical trials in the Latin American and Caribbean Health Science Literature database (LILACS database): update. Sao Paulo Med J.

[28] Egger M, Smith GD, Altman D (2001). Systematic reviews in health care.

[29] Review Manager (RevMan). [Computer program]. Version 5.1. Copenhagen: The Nordic Cochrane Centre, The Cochrane Collaboration, 2011.

[30] Parmar MK, Torri V, Stewart L (1998). Extracting summary statistics to perform meta-analyses of the published literature for survival endpoints. Stat Med.

[31] Higgins JP, Thompson SG, Deeks JJ, Altman DG (2003). Measuring inconsistency in meta-analyses. BMJ.

[32] Yang K, Wang YJ, Chen XR, Chen HN (2010). Effectiveness and safety of bevacizumab for unresectable non-small-cell lung cancer: a meta-analysis. Clin Drug Investig.

[33] Deeks JJ, Higgins JP, Altman DG, Higgins JPGS (2006). Analysing and presenting results. Cochrane handbook for systematic reviews of in- terventions (ed 426 [updated September 2006]).

[34] DerSimonian R, Laird N (1986). Meta-analysis in clinical trials. Control Clin Trials.

[35] Egger M, Davey Smith G, Schneider M, Minder C (1997). Bias in meta-analysis detected by a simple, graphical test. BMJ.

[36] McQuay HJ, Moore RA (1997). Using numerical results from systematic reviews in clinical practice. Ann Intern Med.

[37] Smeeth L, Haines A, Ebrahim S (1999). Numbers needed to treat derived from meta-analyses--sometimes informative, usually misleading. BMJ.

[38] Altman DG, Deeks JJ (2002). Meta-analysis, Simpson’s paradox, and the number needed to treat. BMC Med Res Methodol.

[39] Liberati A, Altman DG, Tetzlaff J, Mulrow C, Gotzsche PC, Ioannidis JP (2009). The PRISMA statement for reporting systematic reviews and meta-analyses of studies that evaluate health care interventions: explanation and elaboration. Ann Intern Med.

[40] Tebbutt NC, Wilson K, Gebski VJ, Cummins MM, Zannino D, van Hazel GA (2010). Capecitabine, bevacizumab, and mitomycin in first-line treatment of metastatic colorectal cancer: results of the Australasian Gastrointestinal Trials Group Randomized Phase III MAX Study. J Clin Oncol.

[41] Price TJ, Hardingham JE, Lee CK, Weickhardt A, Townsend AR, Wrin JW (2011). Impact of KRAS and BRAF gene mutation status on outcomes from the phase III AGITG MAX trial of capecitabine alone or in combination with Bevacizumab and mitomycin in advanced colorectal cancer. J Clin Oncol.

[42] Hurwitz HI, Fehrenbacher L, Hainsworth JD, Heim W, Berlin J, Holmgren E (2005). Bevacizumab in combination with fluorouracil and leucovorin: an active regimen for first-line metastatic colorectal cancer. J Clin Oncol.

[43] Hurwitz HI, Yi J, Ince W, Novotny WF, Rosen O (2009). The clinical benefit of bevacizumab in metastatic colorectal cancer is independent of K-ras mutation status: analysis of a phase III study of bevacizumab with chemotherapy in previously untreated metastatic colorectal cancer. Oncologist.

[44] Stathopoulos GP, Batziou C, Trafalis D, Koutantos J, Batzios S, Stathopoulos J (2010). Treatment of colorectal cancer with and without bevacizumab: a phase III study. Oncology.

[45] Cassidy J, Clarke S, Diaz-Rubio E, Scheithauer W, Figer A, Wong R (2011). XELOX vs FOLFOX-4 as first-line therapy for metastatic colorectal cancer: NO16966 updated results. Br J Cancer.

[46] Saltz LB, Clarke S, Diaz-Rubio E, Scheithauer W, Figer A, Wong R (2008). Bevacizumab in combination with oxaliplatin-based chemotherapy as first-line therapy in metastatic colorectal cancer: a randomized phase III study. J Clin Oncol.

[47] Kabbinavar F, Hurwitz HI, Fehrenbacher L, Meropol NJ, Novotny WF, Lieberman G (2003). Phase II, randomized trial comparing bevacizumab plus fluorouracil (FU)/leucovorin (LV) with FU/LV alone in patients with metastatic colorectal cancer. J Clin Oncol.

[48] Kabbinavar FF, Schulz J, McCleod M, Patel T, Hamm JT, Hecht JR (2005). Addition of bevacizumab to bolus fluorouracil and leucovorin in first-line metastatic colorectal cancer: results of a randomized phase II trial. J Clin Oncol.

[49] Chari RS, Helton WS, Marsh RD (2006). Chemotherapy and regional therapy of hepatic colorectal metastases: expert consensus statement by Bartlett et al. Ann Surg Oncol.

[50] Glimelius B, Oliveira J (2008). Rectal cancer: ESMO clinical recommendations for diagnosis, treatment and follow-up. Ann Oncol.

[51] Glimelius B, Oliveira J (2009). Rectal cancer: ESMO clinical recommendations for diagnosis, treatment and follow-up. Ann Oncol.

[52] Nordlinger B, Van Cutsem E, Gruenberger T, Glimelius B, Poston G, Rougier P (2009). Combination of surgery and chemotherapy and the role of targeted agents in the treatment of patients with colorectal liver metastases: recommendations from an expert panel. Ann Oncol.

[53] Nordlinger B, Van Cutsem E, Rougier P, Kohne CH, Ychou M, Sobrero A (2007). Does chemotherapy prior to liver resection increase the potential for cure in patients with metastatic colorectal cancer? A report from the European Colorectal Metastases Treatment Group. Eur J Cancer (Oxford, England : 1990).

[54] Papamichael D, Audisio R, Horiot JC, Glimelius B, Sastre J, Mitry E (2009). Treatment of the elderly colorectal cancer patient: SIOG expert recommendations. Ann Oncol.

[55] Van Cutsem EJ, Oliveira J (2008). Advanced colorectal cancer: ESMO clinical recommendations for diagnosis, treatment and follow-up. Ann Oncol.

[56] Edwards MS, Chadda SD, Zhao Z, Barber BL, Sykes DP (2012). A systematic review of treatment guidelines for metastatic colorectal cancer. Colorectal Dis.

[57] Aranda E, Aparicio J, Alonso V, Garcia-Albeniz X, Garcia-Alfonso P, Salazar R (2015). SEOM clinical guidelines for diagnosis and treatment of metastatic colorectal cancer 2015. Clinical & translational oncology : official publication of the Federation of Spanish Oncology Societies and of the National Cancer Institute of Mexico.

[58] Van Cutsem E, Cervantes A, Nordlinger B, Arnold D (2014). Metastatic colorectal cancer: ESMO Clinical Practice Guidelines for diagnosis, treatment and follow-up. Annals of oncology : official journal of the European Society for Medical Oncology/ESMO.

[59] Watanabe T, Itabashi M, Shimada Y, Tanaka S, Ito Y, Ajioka Y (2015). Japanese Society for Cancer of the Colon and Rectum (JSCCR) Guidelines 2014 for treatment of colorectal cancer. Int J Clin Oncol.

[60] National Institute for Health and Care Excellence (NICE). Colorectal cancer: diagnosis and management. Last updated: december 2014. http://www.nice.org.uk.

[61] Lv C, Wu S, Zheng D, Wu Y, Yao D, Yu X (2013). The efficacy of additional bevacizumab to cytotoxic chemotherapy regimens for the treatment of colorectal cancer: an updated meta-analysis for randomized trials. Cancer Biother Radiopharm.

[62] Hu W, Xu W, Liao X, He H. Bevacizumab in combination with first-line chemotherapy in patients with metastatic colorectal cancer: a meta-analysis. Minerva Chir. 2015;27. 26013763

[63] Qu CY, Zheng Y, Zhou M, Zhang Y, Shen F, Cao J (2015). Value of Bevacizumab in treatment of colorectal cancer: a meta-analysis. World J Gastroenterol.

[64] Zhang G, Zhou X, Lin C (2015). Efficacy of chemotherapy plus bevacizumab as first-line therapy in patients with metastatic colorectal cancer: a meta-analysis and up-date. Int J Clin Exp Med.

[65] Giantonio BJ, Catalano PJ, Meropol NJ, O’Dwyer PJ, Mitchell EP, Alberts SR (2007). Bevacizumab in combination with oxaliplatin, fluorouracil, and leucovorin (FOLFOX4) for previously treated metastatic colorectal cancer: results from the Eastern Cooperative Oncology Group Study E3200. J Clin Oncol.

[66] Buyse M, Burzykowski T, Carroll K, Michiels S, Sargent DJ, Miller LL (2007). Progression-free survival is a surrogate for survival in advanced colorectal cancer. J Clin Oncol.

[67] Giessen C, Laubender RP, Ankerst DP, Stintzing S, Modest DP, Mansmann U (2013). Progression-free survival as a surrogate endpoint for median overall survival in metastatic colorectal cancer: literature-based analysis from 50 randomized first-line trials. Clin Cancer Res.

[68] Ferrara N, Davis-Smyth T (1997). The biology of vascular endothelial growth factor. Endocr Rev.

[69] Jubb AM, Pham TQ, Hanby AM, Frantz GD, Peale FV, Wu TD (2004). Expression of vascular endothelial growth factor, hypoxia inducible factor 1alpha, and carbonic anhydrase IX in human tumours. J Clin Pathol.

[70] Lima JP, Rodrigues DN, Carcano FM, Cruz MR, dos Santos LV, editors. VEGF-A level is a predictor of bevacizumab benefit for breast cancer: results of meta-analysis. European Society for Medical Oncology - ESMO 2014, abst 374P.

[71] Botrel TE, Clark O, Clark L, Paladini L, Faleiros E, Pegoretti B. Efficacy of bevacizumab (Bev) plus chemotherapy (CT) compared to CT alone in previously untreated locally advanced or metastatic non-small cell lung cancer (NSCLC): systematic review and meta-analysis. Lung Cancer (Amsterdam, Netherlands). 2011;74(1):89–97. 10.1016/j.lungcan.2011.01.028 21377753

[72] Lee EK, Revil C, Ngoh CA, Lister J, Kwon JM, Park MH (2012). Clinical and cost effectiveness of bevacizumab + FOLFIRI combination versus FOLFIRI alone as first-line treatment of metastatic colorectal cancer in South Korea. Clin Ther.

[73] Shiroiwa T, Fukuda T, Tsutani K (2010). Out-of-pocket payment and cost-effectiveness of XELOX and XELOX plus bevacizumab therapy: from the perspective of metastatic colorectal cancer patients in Japan. Int J Clin Oncol.

[74] Zhang H, Xu L, An G (2012). Evaluation of bevacizumab combined with FOLFIRI as first-line treatment for patients with metastatic colorectal cancer. Cancer Res Pre Treat.

[75] Allegra CJ, Yothers G, O’Connell MJ, Sharif S, Colangelo LH, Lopa SH (2009). Initial safety report of NSABP C-08: a randomized phase III study of modified FOLFOX6 with or without Bevacizumab for the adjuvant treatment of patients with stage II or III colon cancer. J Clin Oncol.

[76] Allegra CJ, Yothers G, O’Connell MJ, Sharif S, Petrelli NJ, Colangelo LH (2011). Phase III trial assessing bevacizumab in stages II and III carcinoma of the colon: results of NSABP protocol C-08. J Clin Oncol.

[77] Ducreux M, Adenis A, Mendiboure J, Francois E, Boucher E, Chauffert B, et al. Efficacy and safety of bevacizumab (BEV)-based combination regimens in patients with metastatic colorectal cancer (mCRC): ran- domized phase II study of BEV # FOLFIRI versus BEV # XELIRI (FNCLCC ACCORD 13/0503 study). J Clin Oncol. 2009;27(153):suppl; abstra 4086.

[78] Pectasides D, Papaxoinis G, Kalogeras KT, Eleftheraki AG, Xanthakis I, Makatsoris T (2012). XELIRI-bevacizumab versus FOLFIRI-bevacizumab as first-line treatment in patients with metastatic colorectal cancer: a Hellenic Cooperative Oncology Group phase III trial with collateral biomarker analysis. BMC Cancer.

[79] Souglakos J, Ziras N, Kakolyris S, Boukovinas I, Kentepozidis N, Makrantonakis P (2012). Randomised phase-II trial of CAPIRI (capecitabine, irinotecan) plus bevacizumab vs FOLFIRI (folinic acid, 5-fluorouracil, irinotecan) plus bevacizumab as first-line treatment of patients with unresectable/metastatic colorectal cancer (mCRC). Br J Cancer.

[80] Diaz-Rubio E, Gomez-Espana A, Massuti B, Sastre J, Abad A, Valladares M (2012). First-line XELOX plus bevacizumab followed by XELOX plus bevacizumab or single-agent bevacizumab as maintenance therapy in patients with metastatic colorectal cancer: the phase III MACRO TTD study. Oncologist.

[81] Price TJ, Zannino D, Wilson K, Simes RJ, Cassidy J, Van Hazel GA (2012). Bevacizumab is equally effective and no more toxic in elderly patients with advanced colorectal cancer: a subgroup analysis from the AGITG MAX trial: an international randomised controlled trial of Capecitabine, Bevacizumab and Mitomycin C. Ann Oncol.

[82] Moehler M, Sprinzl MF, Abdelfattah M, Schimanski CC, Adami B, Godderz W (2009). Capecitabine and irinotecan with and without bevacizumab for advanced colorectal cancer patients. World J Gastroenterol.

