# Supplementary material for: Efficacy and safety of bevacizumab plus chemotherapy compared to chemotherapy alone in previously untreated advanced or metastatic colorectal cancer: a systematic review and meta-analysis
Source: BMC Cancer. 2016 Aug 24;16(1):677. doi: 10.1186/s12885-016-2734-y (PMC4997727; doi:10.1186/s12885-016-2734-y)
Supplement: Additional file 1: — Quality assessment (risk of bias) of randomized studies evaluating bevacizumab plus chemotherapy in patients with mCRC in first line chemotherapy. (DOCX 46 kb) [file 12885_2016_2734_MOESM1_ESM.docx]

| **Additional file 1 - Quality assessment (risk of bias) of randomized studies evaluating bevacizumab plus chemotherapy in patients with mCRC in first line chemotherapy** | | | | | | | |
| --- | --- | --- | --- | --- | --- | --- | --- |
| Study | Random sequence generation | Allocation concealment | Blinding of participants and personnel | Blinding of outcome assessment | Incomplete outcome data | Selective reporting | Other bias |
| Hurwitz 2004/2005 ([12](#_ENREF_12), [36](#_ENREF_36)) (AVF 2107) | low risk | low risk | low risk | low risk | low risk | uncertain | uncertain |
| Guan 2011 ([37](#_ENREF_37)) (ARTIST) | uncertain | uncertain | low risk | low risk | low risk | uncertain | uncertain |
| Stathopoulos 2010 ([38](#_ENREF_38)) | uncertain | uncertain | uncertain | uncertain | low risk | uncertain | uncertain |
| Saltz / Cassidy 2008/2011 ([39](#_ENREF_39), [40](#_ENREF_40)) (NO16966) | low risk | low risk | uncertain | uncertain | low risk | uncertain | uncertain |
| Passardi 2013/2015 ([18](#_ENREF_18), [20](#_ENREF_20)) (ITACA)** | low risk | low risk | uncertain | uncertain | low risk | uncertain | uncertain |
| Kabinnavar 2003 ([41](#_ENREF_41)) | uncertain | uncertain | low risk | low risk | low risk | uncertain | high risk |
| Kabinnavar 2005 ([42](#_ENREF_42)) | low risk | low risk | low risk | low risk | low risk | uncertain | high risk |
| Tebutt 2010 ([43](#_ENREF_43)) (MAX) | uncertain | uncertain | low risk | low risk | low risk | uncertain | uncertain |
| Cunningham 2013 ([19](#_ENREF_19)) (AVEX) | low risk | low risk | low risk | low risk | low risk | uncertain | uncertain |
