# Supplementary material for: Efficacy and safety of bevacizumab plus chemotherapy compared to chemotherapy alone in previously untreated advanced or metastatic colorectal cancer: a systematic review and meta-analysis
Source: BMC Cancer. 2016 Aug 24;16(1):677. doi: 10.1186/s12885-016-2734-y (PMC4997727; doi:10.1186/s12885-016-2734-y)
Supplement: Additional file 2: Figure S1. — Comparative effect in hematologic toxicities of chemotherapy with bevacizumab versus chemotherapy alone. (PDF 503 kb) [file 12885_2016_2734_MOESM2_ESM.pdf]

Study or Subgroup	CT with Bevacizumab Events	CT with Bevacizumab Total	CT alone Events	CT alone Total	Weight	Risk Ratio M-H, Fixed, 95% CI
**4.1.1 Anemia**						
Cunningham 2013	0	134	0	136		Not estimable
Guan 2011	6	141	1	70	21.9%	2.98 [0.37, 24.26]
Hurwitz 2004/2005	0	393	0	397		Not estimable
Kabbinavar 2003	0	67	0	35		Not estimable
Kabbinavar 2005	0	100	0	104		Not estimable
Passardi 2013/2015	2	176	5	194	78.1%	0.44 [0.09, 2.24]
Saltz/Cassidy 2008/2011	0	694	0	675		Not estimable
Stathopoulos 2010	0	114	0	108		Not estimable
Tebutt 2010	0	157	0	156		Not estimable
**Subtotal (95% CI)**		**1976**		**1875**	**100.0%**	**1.00 [0.31, 3.18]**
Total events	8		6			
Heterogeneity: $Chi^2 = 2.01$, df = 1 (P = 0.16); $I^2 = 50\%$						
Test for overall effect: Z = 0.00 (P = 1.00)						
**4.1.2 Neutropenia**						
Cunningham 2013	1	134	1	136	0.4%	1.01 [0.06, 16.06]
Guan 2011	47	141	13	70	7.6%	1.79 [1.04, 3.09]
Hurwitz 2004/2005	145	393	123	397	53.3%	1.19 [0.98, 1.45]
Kabbinavar 2003	3	67	1	35	0.6%	1.57 [0.17, 14.52]
Kabbinavar 2005	5	100	7	104	3.0%	0.74 [0.24, 2.26]
Passardi 2013/2015	69	176	82	194	34.0%	0.93 [0.72, 1.19]
Saltz/Cassidy 2008/2011	0	694	0	675		Not estimable
Stathopoulos 2010	0	114	0	108		Not estimable
Tebutt 2010	0	157	2	156	1.1%	0.20 [0.01, 4.11]
**Subtotal (95% CI)**		**1976**		**1875**	**100.0%**	**1.12 [0.97, 1.30]**
Total events	270		229			
Heterogeneity: $Chi^2 = 7.40$, df = 6 (P = 0.29); $I^2 = 19\%$						
Test for overall effect: Z = 1.57 (P = 0.12)						
**4.1.3 Febrile neutropenia**						
Cunningham 2013	0	134	0	136		Not estimable
Guan 2011	3	141	2	70	28.2%	0.74 [0.13, 4.35]
Hurwitz 2004/2005	0	393	0	397		Not estimable
Kabbinavar 2003	0	67	0	35		Not estimable
Kabbinavar 2005	0	100	0	104		Not estimable
Passardi 2013/2015	1	176	4	194	40.1%	0.28 [0.03, 2.44]
Saltz/Cassidy 2008/2011	0	694	0	675		Not estimable
Stathopoulos 2010	0	114	0	108		Not estimable
Tebutt 2010	4	157	3	156	31.7%	1.32 [0.30, 5.82]
**Subtotal (95% CI)**		**1976**		**1875**	**100.0%**	**0.74 [0.28, 1.94]**
Total events	8		9			
Heterogeneity: $Chi^2 = 1.38$, df = 2 (P = 0.50); $I^2 = 0\%$						
Test for overall effect: Z = 0.61 (P = 0.54)						
**4.1.4 Trombocytopenia**						
Cunningham 2013	0	134	0	136		Not estimable
Guan 2011	4	141	3	70	67.8%	0.66 [0.15, 2.88]
Hurwitz 2004/2005	0	393	0	397		Not estimable
Kabbinavar 2003	0	67	0	35		Not estimable
Kabbinavar 2005	0	100	0	104		Not estimable
Passardi 2013/2015	4	176	2	194	32.2%	2.20 [0.41, 11.89]
Saltz/Cassidy 2008/2011	0	694	0	675		Not estimable
Stathopoulos 2010	0	114	0	108		Not estimable
Tebutt 2010	0	157	0	156		Not estimable
**Subtotal (95% CI)**		**1976**		**1875**	**100.0%**	**1.16 [0.40, 3.35]**
Total events	8		5			
Heterogeneity: $Chi^2 = 1.12$, df = 1 (P = 0.29); $I^2 = 11\%$						
Test for overall effect: Z = 0.27 (P = 0.79)						

Test for subgroup differences: $Chi^2 = 0.74$, df = 3 (P = 0.86), $I^2 = 0\%$

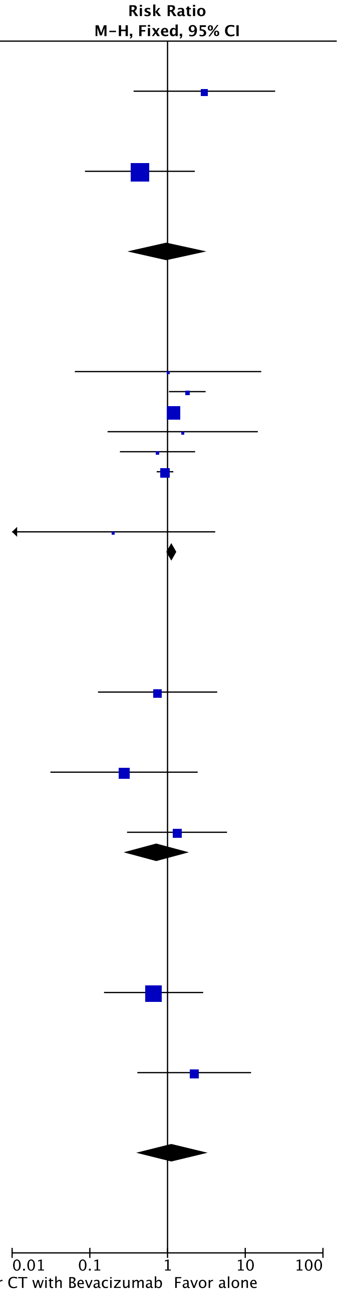

Figure 6. Comparative effect in hematologic toxicities of chemotherapy with bevacizumab versus chemotherapy alone

Abbreviations: CT, chemotherapy; CI, confidence interval
