# Supplementary material for: Efficacy and safety of bevacizumab plus chemotherapy compared to chemotherapy alone in previously untreated advanced or metastatic colorectal cancer: a systematic review and meta-analysis
Source: BMC Cancer. 2016 Aug 24;16(1):677. doi: 10.1186/s12885-016-2734-y (PMC4997727; doi:10.1186/s12885-016-2734-y)
Supplement: Additional file 3: Figure S2. — Comparative effect in non-hematologic toxicities of chemotherapy with bevacizumab versus chemotherapy alone. (PDF 864 kb) [file 12885_2016_2734_MOESM3_ESM.pdf]

Study or Subgroup	CT with Bevacizumab Events	CT with Bevacizumab Total	CT alone Events	CT alone Total	Weight	Risk Ratio M-H, Fixed, 95% CI
**4.2.1 Diarrhea**						
Cunningham 2013	9	134	9	136	4.2%	1.01 [0.42, 2.48]
Guan 2011	37	141	15	70	9.4%	1.22 [0.72, 2.07]
Hurwitz 2004/2005	127	393	98	397	45.9%	1.31 [1.05, 1.64]
Kabbinavar 2003	20	67	13	35	8.0%	0.80 [0.46, 1.42]
Kabbinavar 2005	39	100	42	104	19.4%	0.97 [0.69, 1.35]
Passardi 2013/2015	14	176	11	194	4.9%	1.40 [0.65, 3.01]
Saltz/Cassidy 2008/2011	0	694	0	675		Not estimable
Stathopoulos 2010	0	114	0	108		Not estimable
Tebutt 2010	27	157	17	156	8.0%	1.58 [0.90, 2.78]
**Subtotal (95% CI)**		**1976**		**1875**	**100.0%**	**1.21 [1.03, 1.41]**
Total events	273		205			
Heterogeneity: Chi^2 = 5.32, df = 6 (P = 0.50); I^2 = 0%						
Test for overall effect: Z = 2.37 (P = 0.02)						
**4.2.2 Hypertension**						
Cunningham 2013	3	134	2	136	4.5%	1.52 [0.26, 8.97]
Guan 2011	6	141	0	70	1.5%	6.50 [0.37, 113.76]
Hurwitz 2004/2005	43	393	9	397	20.2%	4.83 [2.39, 9.77]
Kabbinavar 2003	11	67	0	35	1.5%	12.18 [0.74, 200.73]
Kabbinavar 2005	16	100	3	104	6.6%	5.55 [1.67, 18.46]
Passardi 2013/2015	49	176	21	194	45.1%	2.57 [1.61, 4.11]
Saltz/Cassidy 2008/2011	26	694	8	675	18.3%	3.16 [1.44, 6.93]
Stathopoulos 2010	0	114	0	108		Not estimable
Tebutt 2010	6	157	1	156	2.3%	5.96 [0.73, 48.95]
**Subtotal (95% CI)**		**1976**		**1875**	**100.0%**	**3.56 [2.58, 4.92]**
Total events	160		44			
Heterogeneity: Chi^2 = 5.20, df = 7 (P = 0.64); I^2 = 0%						
Test for overall effect: Z = 7.73 (P < 0.00001)						
**4.2.3 Proteinuria**						
Cunningham 2013	2	134	0	136	1.6%	5.07 [0.25, 104.71]
Guan 2011	1	141	0	70	2.2%	1.50 [0.06, 36.36]
Hurwitz 2004/2005	3	393	3	397	9.7%	1.01 [0.21, 4.97]
Kabbinavar 2003	0	67	0	35		Not estimable
Kabbinavar 2005	1	100	0	104	1.6%	3.12 [0.13, 75.67]
Passardi 2013/2015	39	176	26	194	80.1%	1.65 [1.05, 2.60]
Saltz/Cassidy 2008/2011	4	694	0	675	1.6%	8.75 [0.47, 162.28]
Stathopoulos 2010	0	114	0	108		Not estimable
Tebutt 2010	5	157	1	156	3.2%	4.97 [0.59, 42.04]
**Subtotal (95% CI)**		**1976**		**1875**	**100.0%**	**1.89 [1.26, 2.84]**
Total events	55		30			
Heterogeneity: Chi^2 = 3.30, df = 6 (P = 0.77); I^2 = 0%						
Test for overall effect: Z = 3.07 (P = 0.002)						
**4.2.4 Gastrointestinal perforation**						
Cunningham 2013	0	134	0	136		Not estimable
Guan 2011	1	141	0	70	14.2%	1.50 [0.06, 36.36]
Hurwitz 2004/2005	6	393	0	397	10.6%	13.13 [0.74, 232.32]
Kabbinavar 2003	0	67	0	35		Not estimable
Kabbinavar 2005	2	100	0	104	10.5%	5.20 [0.25, 106.95]
Passardi 2013/2015	0	176	0	194		Not estimable
Saltz/Cassidy 2008/2011	4	694	2	675	43.3%	1.95 [0.36, 10.59]
Stathopoulos 2010	0	114	0	108		Not estimable
Tebutt 2010	3	157	1	156	21.4%	2.98 [0.31, 28.35]
**Subtotal (95% CI)**		**1976**		**1875**	**100.0%**	**3.63 [1.31, 10.09]**
Total events	16		3			
Heterogeneity: Chi^2 = 1.67, df = 4 (P = 0.80); I^2 = 0%						
Test for overall effect: Z = 2.47 (P = 0.01)						
**4.2.5 Nausea /vomiting**						
Cunningham 2013	4	134	2	136	5.5%	2.03 [0.38, 10.90]
Guan 2011	18	141	8	70	29.4%	1.12 [0.51, 2.44]
Hurwitz 2004/2005	0	393	0	397		Not estimable
Kabbinavar 2003	0	67	0	35		Not estimable
Kabbinavar 2005	0	100	0	104		Not estimable
Passardi 2013/2015	9	176	7	194	18.3%	1.42 [0.54, 3.73]
Saltz/Cassidy 2008/2011	0	694	0	675		Not estimable
Stathopoulos 2010	0	114	0	108		Not estimable
Tebutt 2010	16	157	17	156	46.9%	0.94 [0.49, 1.78]
**Subtotal (95% CI)**		**1976**		**1875**	**100.0%**	**1.14 [0.74, 1.74]**
Total events	47		34			
Heterogeneity: Chi^2 = 1.01, df = 3 (P = 0.80); I^2 = 0%						
Test for overall effect: Z = 0.59 (P = 0.56)						
**4.2.6 Any thromboembolic events**						
Cunningham 2013	16	134	7	136	4.2%	2.32 [0.99, 5.46]
Guan 2011	1	141	0	70	0.4%	1.50 [0.06, 36.36]
Hurwitz 2004/2005	76	393	64	397	38.2%	1.20 [0.89, 1.62]
Kabbinavar 2003	7	67	1	35	0.8%	3.66 [0.47, 28.55]
Kabbinavar 2005	18	100	19	104	11.2%	0.99 [0.55, 1.77]
Passardi 2013/2015	37	176	25	194	14.3%	1.63 [1.03, 2.60]
Saltz/Cassidy 2008/2011	66	694	40	675	24.3%	1.60 [1.10, 2.34]
Stathopoulos 2010	0	114	0	108		Not estimable
Tebutt 2010	19	157	11	156	6.6%	1.72 [0.84, 3.49]
**Subtotal (95% CI)**		**1976**		**1875**	**100.0%**	**1.44 [1.20, 1.73]**
Total events	240		167			
Heterogeneity: Chi^2 = 5.84, df = 7 (P = 0.56); I^2 = 0%						
Test for overall effect: Z = 3.86 (P = 0.0001)						
**4.2.7 Bleeding**						
Cunningham 2013	0	134	1	136	4.0%	0.34 [0.01, 8.23]
Guan 2011	1	141	1	70	3.6%	0.50 [0.03, 7.82]
Hurwitz 2004/2005	12	393	10	397	26.8%	1.21 [0.53, 2.77]
Kabbinavar 2003	3	67	0	35	1.8%	3.71 [0.20, 69.79]
Kabbinavar 2005	5	100	3	104	7.9%	1.73 [0.43, 7.06]
Passardi 2013/2015	30	176	9	194	23.1%	3.67 [1.79, 7.52]
Saltz/Cassidy 2008/2011	13	694	8	675	21.9%	1.58 [0.66, 3.79]
Stathopoulos 2010	0	114	0	108		Not estimable
Tebutt 2010	2	157	4	156	10.8%	0.50 [0.09, 2.67]
**Subtotal (95% CI)**		**1976**		**1875**	**100.0%**	**1.81 [1.22, 2.67]**
Total events	66		36			
Heterogeneity: Chi^2 = 9.15, df = 7 (P = 0.24); I^2 = 24%						
Test for overall effect: Z = 2.97 (P = 0.003)						

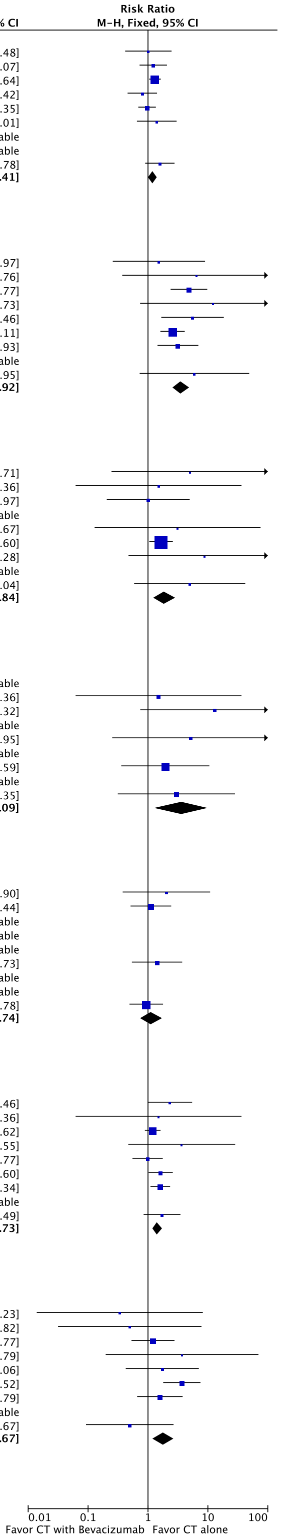

Test for subgroup differences: Chi^2 = 41.99, df = 6 (P < 0.00001), I^2 = 85.7%

Figure 7. Comparative effect in non-hematologic toxicities of chemotherapy with bevacizumab versus chemotherapy alone

Abbreviations: CT, chemotherapy; CI, confidence interval
