# Supplementary material for: Efficacy and safety of bevacizumab plus chemotherapy compared to chemotherapy alone in previously untreated advanced or metastatic colorectal cancer: a systematic review and meta-analysis
Source: BMC Cancer. 2016 Aug 24;16(1):677. doi: 10.1186/s12885-016-2734-y (PMC4997727; doi:10.1186/s12885-016-2734-y)
Supplement: Additional file 4: — Assessment of publication bias. Funnel plot for objective response rates, progression-free survival and overall survival in this meta-analysis. (DOCX 352 kb) [file 12885_2016_2734_MOESM4_ESM.docx]

Assessment of publication bias. Funnel plot for objective response rates, progression-free survival and overall survival in this meta-analysis.

1 - Objective response rates: All studies are evenly distributed on both sides, thus presenting basic symmetry and indicating that there was no publication bias.

2 - Progression-free survival: All studies are evenly distributed on both sides, thus presenting basic symmetry and indicating that there was no publication bias.

3 - Overall survival: All studies are evenly distributed on both sides, thus presenting basic symmetry and indicating that there was no publication bias.
